# Twisted Nanographenes with Robust Conformational Stability

**DOI:** 10.3390/nano14211737

**Published:** 2024-10-30

**Authors:** Penghui Song, Yoshifumi Hashikawa, Chaolumen

**Affiliations:** 1 College of Chemistry and Chemical Engineering, Inner Mongolia University, Hohhot 010021, China; 2 Institute for Chemical Research, Kyoto University, Uji 611-0011, Kyoto, Japan

**Keywords:** nanographene, twist, helicene

## Abstract

Owing to a lack of methodology for rationally and selectively synthesizing twisted nanographenes, it is usually inevitable that we obtain nanographenes as a mixture with various geometries, such as unidirectionally twisted, alternatively twisted, randomly twisted, and even wavy structures, reflecting the high activation barriers among them. Otherwise, they are interconvertible if the barriers are low enough such that only averaged properties can be observed under a thermal equilibrium. Recently, we reported on a double-twisted nanographene containing four [6]helicene units within the skeleton. In this paper, we discuss the robust conformational stability of the nanographene, both experimentally and computationally. The results indicate that the nanographene could only be racemized at temperatures exceeding 200 °C, and the first flip of one of the four [6]helicene units is the rate-degerming step.

## 1. Introduction

The topological structures of nanocarbons have become more and more diversified in recent decades [1,2,3,4,5,6,7,8,9]. Among them, the synthesis of chiral nanographenes (NGs) is a topic of interest in targeting chiroptical switches, chiral sensors, spin filters, and so forth [10,11], wherein (multiple) helical NGs, including π-expanded helicenes, have been most extensively studied [12,13]; while NGs possessing axial [14,15] or twist [16,17,18,19,20,21,22,23] chirality have been less explored. This is probably because of the difficulty in placing suitably bulky and large substituents, which potentially prevent the axial rotation of the two NG surfaces, in axial chiral NGs, as well as in specifying a contorted geometry during, or even after, the synthesis of twisted NGs.

**Figure 1:**
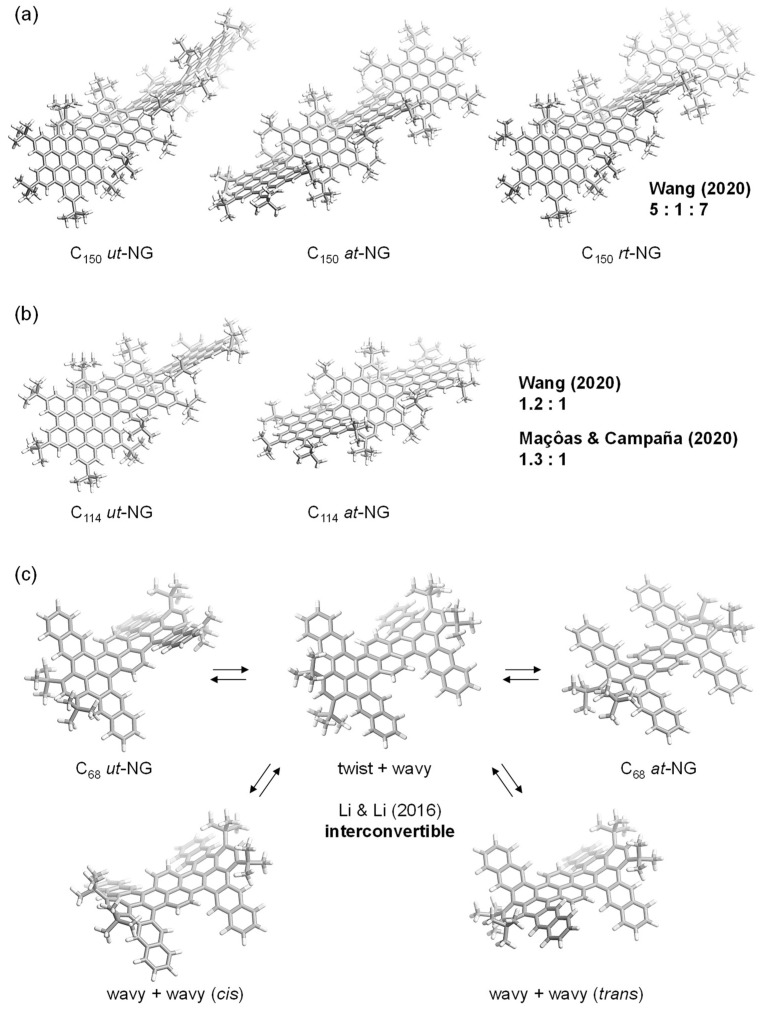
Representative examples of twisted nanographenes: (**a**) C_150_ NGs; (**b**) C_114_ NGs; and (**c**) C_68_ NGs [24,25,26].

In fact, Wang and co-workers reported in 2020 that the synthesis of C_150_ supertwistacene—that is, unidirectionally twisted NG (*ut*-NG)—was accomplished concomitantly with the generation of two other NGs with alternatively twisted (*at*-NG) and randomly twisted (*rt*-NG) geometries (Figure 1a) [24]. Because of poor selectivity in the last step (twelve-fold C–C bond formation via Scholl cyclization), these NGs were obtained in low yields of 5 (*ut*-NG), 1 (*at*-NG), and 7% (*rt*-NG), respectively. Using the same strategy, they also synthesized C_114_ NGs, resulting in the generation of two conformers in 31 (*ut*-NG) and 25% (*at*-NG) yields through twelve-fold C–C bond formation (Figure 1b). The synthesis of the C_114_ NGs was independently reported by another group led by Maçôas and Campaña in 2020 [25]. In their study, *ut*-NG and *at*-NG were obtained in a ratio of 1.3:1, with a combined yield of 16%. The generation of multiple conformations is suggestive of the C–C bond formation occurring randomly, and the thus-formed geometries are fixed due to the high racemization barrier of the incorporated [5]helical units. They also synthesized C_115_ NG, in which one hexagon in the C_114_ NG is replaced by cycloheptanone [25]. Though the synthetic methods are comparable to each other, the C_115_ NG was obtained in 33% as the sole conformation, with an alternatively twisted geometry. The high flexibility of C_68_ twisted NGs was also reported by Li and co-workers in 2016 [26]. This NG possesses a four-fold [4]helical unit, which is a cause of the flexibility such that five conformations are interconvertible over 298 K while freezing at 298 K (Figure 1c). As exemplified above, the uncontrollable selectivity of geometry during the synthesis of NGs is a critical obstacle for further research because of the insufficient supply of materials, whereas NGs with structural flexibility cause mixed properties across all possible conformations, rendering their physical origins ambiguous. However, it is difficult to achieve both the preparation of the sole conformation specifically via the synthetic strategy and robust conformational stability because the higher rigidity of NGs is usually a potential cause of generating multiple conformations. Nevertheless, we recently succeeded in synthesizing a double-twisted NG as the sole product, wherein the two twisted geometries are arranged orthogonally through a central pyrene core [27]. In this paper, we discuss its robust conformational stability compared with those of the [6]helicene analogues that are embedded in the NG.

## 2. Results and Discussion

**Figure 2:**
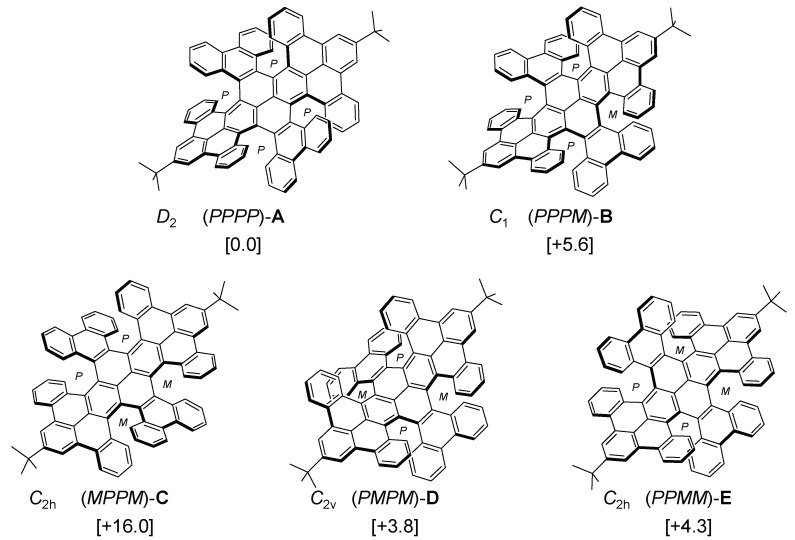
Possible conformations of a double-twisted NG. The Δ*G* values are given in brackets with units in kcal/mol at 298 K (B3LYP-D3/6-31G(d)).

The double-twisted C_76_ NG (Figure 2) was synthesized according to our recent report [28]. This NG possesses four [6]helical units. Therefore, depending upon the arrangement of the stereogenic elements (*P* or *M*), five possible conformations could be considered: (*PPPP*)-**A** (*D*_2_ symmetry); (*PPPM*)-**B** (*C*_1_); (*MPPM*)-**C** (*C*_2h_); (*PMPM*)-**D** (*C*_2v_); and (*PPMM*)-**E** (*C*_2h_). At room temperature, however, this NG exists as the sole conformation, and no other conformational isomers were generated during the synthesis. The crystallographic analysis of the NG revealed its conformation fixed in **A** with *D*_2_ symmetry. According to the theoretical calculations at the B3LYP-D3/6-31G(d) level of theory, **A** is the most stable conformation among the five.

**Figure 3:**
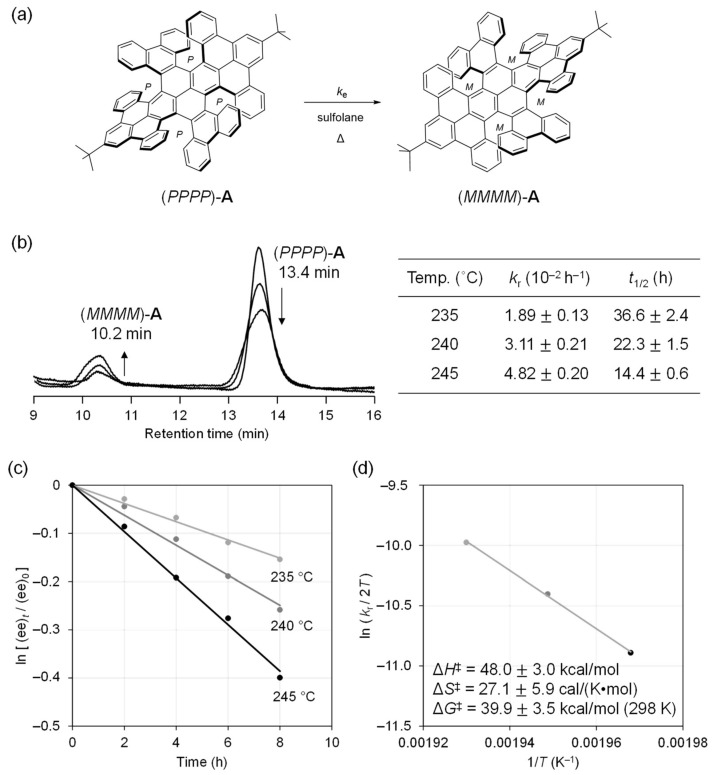
(**a**) Racemization of (*PPPP*)-**A**. (**b**) Chiral HPLC profiles (CHIRALPAK IH-3, CH_2_Cl_2_/MeOH (7:3), r.t., 214 nm) for racemization at 245 °C and kinetic parameters. (**c**) Racemization profiles at 235, 240, and 245 °C with activation barrier *E*_a_. (**d**) Eyring plot with thermodynamic parameters regarding a transition state on the racemization at 298 K.

To verify whether **A** could be transformed into other conformations, we heated a C_2_D_2_Cl_4_ solution of **A** up to 90 °C. However, ^1^H NMR did not show any change, suggesting a high activation barrier and/or the thermodynamic instability of **B**–**E**. To obtain further insights into the rigidity of **A**, we then used (*PPPP*)-**A** to test the racemization in sulfolane (b.p. 285 °C) (Figure 3a). As a result, (*PPPP*)-**A** was found to be racemized over a temperature as high as 235 °C. The racemization process was monitored according to chiral HPLC, showing only two peaks, corresponding to (*PPPP*)-**A** (retention time, 13.4 min) and (*MMMM*)-**A** (10.2 min), without the generation of other conformational isomers, i.e., **B**–**E** (Figure 3b,c). The racemization rates (*k*_r_) were found to be (1.89 ± 0.13) × 10^−2^ (235 °C), (3.11 ± 0.21) × 10^−2^ (240 °C), and (4.82 ± 0.20) × 10^−2^ h^–1^ (245 °C). The half-lives, accordingly, were 36.6 ± 2.4 (235 °C), 22.3 ± 1.5 (240 °C), and 14.4 ± 0.6 h (245 °C). The Eyring plot gave thermodynamic parameters of Δ*G*^‡^ = +39.9 ± 3.5 kcal/mol (298 K), Δ*H*^‡^ = +48.0 ± 3.0 kcal/mol, and Δ*S*^‡^ = +27.1 ± 5.9 cal/(K mol). The Δ*G*^‡^ value is apparently higher than that of [6]helicene (+35.4 kcal/mol) [28], confirming the robust conformational stability of **A**, which originates from the four [6]helical edges fixed around the central pyrene core in close proximity.

**Figure 4:**
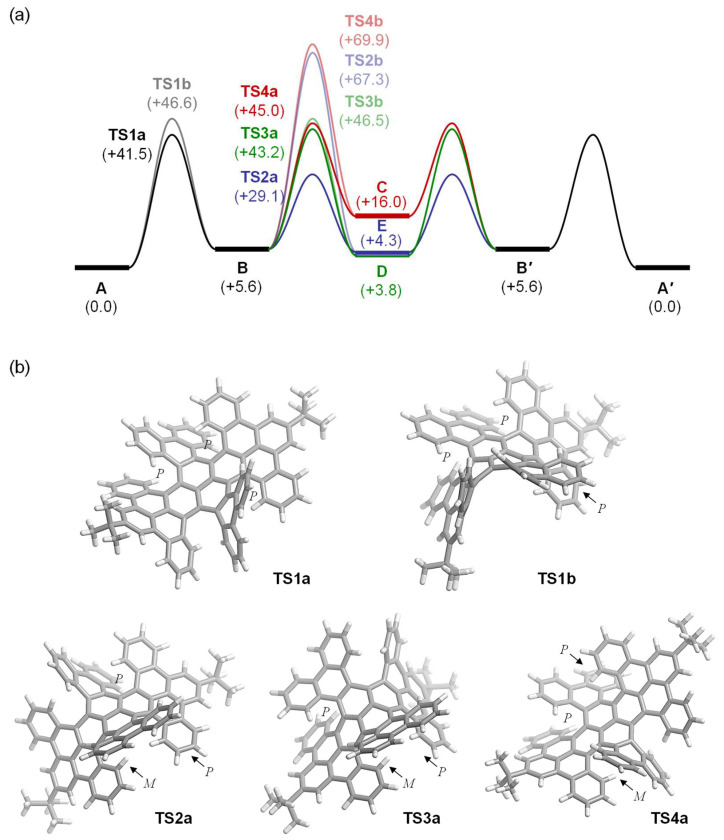
(**a**) Racemization processes from **A** to **A’** (*PPPP* to *MMMM*, for example). (**b**) Optimized structures of selected transition states. Calculations were performed at the B3LYP-D3/6-31G(d) level of theory.

We then examined the racemization process computationally (Figure 4a). The racemization of **A** is considered to be explained by a stepwise process commencing with the conversion of **A** into **B** through the first flip of one of the four [6]helicene units, followed by the second flip of another [6]helicene unit to afford **C**, **D**, or **E**, which then changes into **B’** and consequently gives **A’**, where **A’** and **B’** represent enantiomers of **A** and **B**, respectively. Upon focusing on any of the helical units, one can notice two possible routes for flipping the stereogenic element from *P* to *M* and vice versa, depending on the arrangement of the flipping [6]helicene unit in the +*z* or –*z* direction (**TS1a** and **TS1b**, for instance) perpendicular to the central pyrene plane (Figure 4b). The first flip (**A** → **B**) requires an activation barrier of Δ*G*^‡^ +41.5 kcal/mol (**TS1a**) at 298 K, whilst another route via **TS1b** offers higher value (+46.6 kcal/mol). The second flip then gives **C**, **D**, or **E** with Δ*G*^‡^ + 29.1 (**TS2a**), +43.2 (**TS3a**), and +45.0 (**TS4a**) kcal/mol, respectively. It should be noted that due to the high degree of distortion accumulated within the molecular structures, **TS2b**, **TS3b**, and **TS4b** result in a significant elevation in energy (Δ*G*^‡^ + 67.3, +46.5, and +69.9 kcal/mol, respectively). These activation barriers are hardly climbed under ambient conditions; while, at high temperatures exceeding 200 °C, **A** is converted into **A’**, passing through the route of **A** → **B** → **E** → **B’** → **A’**. Since the second activation barrier (**TS2a**, Δ*G*^‡^ + 29.1 kcal/mol) is smaller by ΔΔ*G*^‡^ − 12.4 kcal/mol than **TS1a** (+41.5 kcal/mol), a rate-determined step is judged to be the first step converting **A** to **B**, and the experimental value (Δ*G*^‡^ = +39.9 ± 3.5 kcal/mol) matched well with that obtained computationally. Importantly, the second-largest barrier was found at the process of **B’** → **A’**, with an energy barrier of Δ*G*^‡^ + 35.9 kcal/mol, which is again smaller than **TS1a** by ΔΔ*G*^‡^ − 5.6 kcal/mol. Therefore, once the first flip of the [6]helicene unit in **A** occurs, **A** is immediately changed into **A’**. This is the reason why we could not observe any other conformations experimentally.

**Figure 5:**
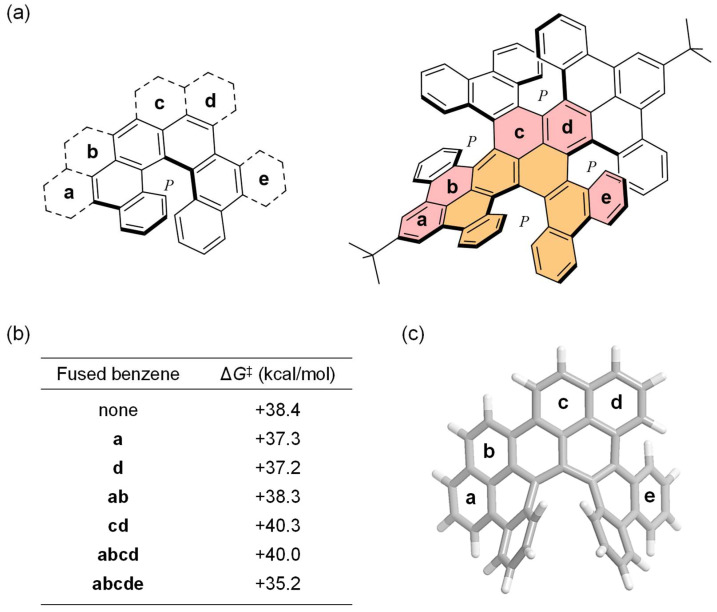
(**a**) Structures of π-expanded [6]helicenes appearing in **A** (orange, [6]helicene; pink, extended benzene rings). (**b**) List of racemization barriers. (**c**) Optimized structure of transition state for **abcde**-fused [6]helicene. Calculations were performed at the B3LYP-D3/6-31G(d) level of theory.

This NG contains a π-expanded [6]helicene unit within its structure. To survey the effect on racemization energies via the peripheral benzo-fusion at the outer rim of the [6]helicene unit, we further examined the racemization of the model structures present in **A** as its segments (Figure 5a). The racemization energy of pristine [6]helicene was calculated to be Δ*G*^‡^ + 38.4 kcal/mol (Figure 5b), which is smaller by ΔΔ*G* − 3.1 kcal/mol than that of **A** (+41.5 kcal/mol). The benzo-fusion at **a** (Δ*G*^‡^ + 37.3 kcal/mol) and **d** (+37.2 kcal/mol) to pristine [6]helicene lowers the activation barriers by ΔΔ*G* ca. −1.0 kcal/mol. The planarization caused by the benzo-fusion at **ab** (Δ*G*^‡^ + 38.3 kcal/mol) does not induce a significant deviation from the barrier of pristine [6]helicene owing to an energetic cancelation of stabilization and repulsion, while the fusion at **cd** (+40.3 kcal/mol) leads to an increase in energy, likely arising from the destabilization of a saddle-shaped transition state. The helix inversion of [6]helicene with fused benzene rings at **abcd** thus requires Δ*G*^‡^ + 40.0 kcal/mol, which is comparable to that of **cd**-fused [6]helicene. Contrastingly, **abcde**-fused [6]helicene offers the smallest energy barrier of +35.2 kcal/mol for its enantiomerization (Figure 5c). This originates from the favorable molecular coordinate induced by the embedded [4]helicene unit comprising four benzene rings containing **d** and **e**. The energy barrier of +41.5 kcal/mol required for the first flip in **A** therefore contains a net increment of ΔΔ*G* + 6.3 kcal/mol relative to **abcde**-fused [6]helicene as a consequence of the distortion offered by the arrangement of the three other [6]helicene units in **A**.

## 3. Methods

The double-twisted C_76_ NG (Figure 2) was synthesized according to our recent report [28]. The kinetic study was performed by using (*PPPP*)-NG in sulfolane at three different temperatures of 235, 240, and 235 °C. The racemization process was monitored every 2 h via chiral HPLC. Chiral HPLC analysis was conducted on a Thermo Surveyor Plus instrument equipped with a CHIRALPAK IH-3 column (4.6 mmφ × 250 mm).

All calculations were performed using the Gaussian 09 program. All structures at the stationery and transition states were optimized at the B3LYP-D3/6-31G(d) level of theory without any symmetry assumptions and confirmed via frequency analysis at the same level of theory, wherein dispersion forces were taken into consideration to improve the reproducibility of the structures.

## 4. Conclusions

In summary, we found robust conformational stability of a double-twisted C_76_ NG whose geometry was fixed in a single stereogenic element at each [6]helical unit (geometry A). Even upon heating at 235 °C, any other conformation could not be observed, while racemization of A slowly proceeded to provide the corresponding enantiomer (A’). From the kinetic study, the activation barrier at 298 K was estimated to be ΔG‡ = +39.9 ± 3.5 kcal/mol, which is larger than pristine [6]helicene (+35.4 kcal/mol), demonstrating robust conformational stability of this NG. Theoretical calculations suggested that the racemization occurs though a route of A → B → E → B’ → A’, wherein the rate-determining step is the first flip of one of the four [6]helicene units in A, with an activation barrier of +41.5 kcal/mol. This value matches well with the experimental results. Since the other steps offer an activation barrier of, at most, ΔG‡ + 35.9 kcal/mol, after claiming the first barrier, A is immediately converted into A’ without there being any chance to observe possible intermediates. In further analysis of the racemization barriers based on π-expanded [6]helicenes appearing in A, the peripheral benzo-annulation to pristine [6]helicene at abcde was suggested to lower the activation barrier to ΔG‡ + 35.2 kcal/mol; therefore, the first flip in A contains a net increment of ΔΔG + 6.3 kcal/mol relative to abcde-fused [6]helicene as a consequence of the distortion offered by the arrangement of the three other [6]helicene units in A.

## Data Availability

All data associated with this manuscript are included in Supplementary Materials.

## Supplementary Materials

The following supporting information can be downloaded at https://www.mdpi.com/article/10.3390/nano14211737/s1: File S1: Molecular coordinates.
