# Supplementary material for: Twisted Nanographenes with Robust Conformational Stability
Source: Nanomaterials (Basel). 2024 Oct 30;14(21):1737. doi: 10.3390/nano14211737 (PMC11547671; doi:10.3390/nano14211737)
Supplement: Supplementary file 1 [file nanomaterials-14-01737-s001.zip › nanomaterials-3260933-supplementary.pdf]

138
A_PPPP_-3233.851703 Hartree

C	7.80141800	0.00164300	0.01377100
C	7.08168500	1.07314800	0.53970000
C	5.67814500	1.09395100	0.57178800
C	7.07553500	-1.06810700	-0.52103700
C	5.67760600	-1.08369300	-0.56699900
C	4.96140700	0.00826000	0.00071200
C	3.51662900	0.00984100	-0.00160900
C	2.81186800	1.17487200	0.39898600
C	3.51802000	2.18080100	1.18764400
C	4.93772600	2.16121200	1.24994700
C	5.59097500	3.14146700	2.02936500
C	4.88499900	4.08836800	2.75049900
C	3.48310700	4.05982200	2.74772200
C	2.81981100	3.11574400	1.98743100
C	0.71718800	0.01016200	0.00077500
C	1.42578200	1.24659800	0.05836500
C	0.69347600	2.48591400	-0.09901500
C	1.31335700	3.73401200	-0.54937000
C	2.51859800	3.73371700	-1.28787600
C	3.05388700	4.90375400	-1.79534300
C	2.39230700	6.12372100	-1.59037100
C	1.19466700	6.14555500	-0.89631700
C	0.63129100	4.96654500	-0.36522200
C	-0.63062900	4.96658300	0.36611800
C	-1.19386700	6.14560300	0.89733800
C	-2.39153500	6.12384000	1.59134700
C	-3.05328600	4.90393600	1.79614200
C	-2.51812800	3.73388900	1.28855800
C	-1.31285700	3.73411700	0.55010300
C	-0.69312400	2.48599400	0.09962000
C	-1.42559700	1.24679500	-0.05790700
C	-2.81166900	1.17528700	-0.39866900
C	-3.51759200	2.18136400	-1.18734300
C	-4.93729500	2.16193200	-1.24990500
C	-5.59029300	3.14252000	-2.02912000
C	-4.88409200	4.08950300	-2.74992300
C	-3.48220500	4.06071500	-2.74702500
C	-2.81915100	3.11636900	-1.98686200
C	9.97059500	1.21510400	0.62580000
C	9.33927600	-0.04340900	0.00232700
C	9.83814300	-0.15785700	-1.45711700
C	9.81946600	-1.27231000	0.80914000
C	-7.80141000	0.00196800	-0.01534800
C	-7.07569900	-1.06766500	0.51993400
C	-5.67780100	-1.08308300	0.56662600
C	-7.08151000	1.07355600	-0.54087000
C	-5.67794800	1.09453700	-0.57220600
C	-4.96140700	0.00886200	-0.00085800
C	-3.51663100	0.01035400	0.00183100
C	-2.81058000	-1.15505900	0.40116400
C	-3.51591500	-2.16190900	1.19103100
C	-4.93511700	-2.14565800	1.25166200
C	-5.58753100	-3.12206400	2.03539900
C	-4.88056100	-4.06534600	2.76092300
C	-3.47897400	-4.03539400	2.75749300
C	-2.81656400	-3.09282600	1.99396000

C	-0.71719100	0.01026000	-0.00029800
C	-1.42605800	-1.22668800	0.05857700
C	-0.69341800	-2.46646700	-0.09856200
C	-1.31413800	-3.71449900	-0.54747900
C	-2.52072500	-3.71443200	-1.28395800
C	-3.05709800	-4.88456300	-1.78992700
C	-2.39539200	-6.10459300	-1.58547900
C	-1.19653900	-6.12630200	-0.89360400
C	-0.63191800	-4.94713600	-0.36404100
C	0.63125200	-4.94717700	0.36493700
C	1.19574100	-6.12635400	0.89461200
C	2.39462500	-6.10472000	1.58643800
C	3.05650100	-4.88475400	1.79071500
C	2.52025500	-3.71461000	1.28464000
C	1.31363300	-3.71460700	0.54821900
C	0.69305800	-2.46654700	0.09918600
C	1.42586700	-1.22688600	-0.05809900
C	2.81037800	-1.15548500	-0.40081700
C	3.51548500	-2.16250900	-1.19066500
C	4.93468400	-2.14641900	-1.25155200
C	5.58684500	-3.12317400	-2.03507200
C	4.87964800	-4.06655500	-2.76024100
C	3.47806700	-4.03636400	-2.75668500
C	2.81590100	-3.09350900	-1.99330100
C	-9.83908300	-0.15800800	1.45420300
C	-9.33926800	-0.04333700	-0.00489900
C	-9.81870900	-1.27225700	-0.81213400
C	-9.97040200	1.21511700	-0.62867900
H	7.62237500	1.91488500	0.94745400
H	7.62378800	-1.91128200	-0.92270700
H	6.67263900	3.14369700	2.09878000
H	5.41872900	4.82695000	3.34237600
H	2.91589400	4.76412100	3.34957800
H	1.73815300	3.07996500	2.02205600
H	3.02076700	2.79399700	-1.48186400
H	3.97699300	4.86960600	-2.36713200
H	2.80243300	7.04503800	-1.99488000
H	0.66565900	7.08650600	-0.78756000
H	-0.66473400	7.08649800	0.78871100
H	-2.80155400	7.04516100	1.99595200
H	-3.97642100	4.86984300	2.36788700
H	-3.02043200	2.79421200	1.48240500
H	-6.67195100	3.14501300	-2.09859900
H	-5.41764400	4.82834800	-3.34163000
H	-2.91481000	4.76505600	-3.34865800
H	-1.73749600	3.08043500	-2.02136900
H	9.67854500	1.33921600	1.67482300
H	11.06280200	1.13405700	0.59398500
H	9.69056200	2.12316000	0.07966700
H	9.50963100	0.70387400	-2.04910500
H	10.93405900	-0.19479100	-1.48454400
H	9.46180800	-1.06221100	-1.94606300
H	9.44226600	-2.21003900	0.38806900
H	10.91508600	-1.32383000	0.80984200
H	9.47895000	-1.21171300	1.84902900
H	-7.62405200	-1.91091700	0.92131100
H	-7.62209600	1.91517900	-0.94898600
H	-6.66925600	-3.12347200	2.10528700

H	-5.41346000	-4.80158700	3.35643200
H	-2.91087100	-4.73690200	3.36176700
H	-1.73491400	-3.05553500	2.02850200
H	-3.02298100	-2.77472800	-1.47774400
H	-3.98117600	-4.85049400	-2.36014600
H	-2.80643100	-7.02603300	-1.98877600
H	-0.66756000	-7.06732100	-0.78538400
H	0.66663900	-7.06731900	0.78651700
H	2.80556100	-7.02616800	1.98982300
H	3.98061200	-4.85074200	2.36088500
H	3.02264500	-2.77494800	1.47828500
H	6.66856300	-3.12483900	-2.10503600
H	5.41236800	-4.80307100	-3.35557100
H	2.90977900	-4.73793200	-3.36071600
H	1.73425300	-3.05606500	-2.02772300
H	-9.51116300	0.70374800	2.04648300
H	-10.93500800	-0.19518800	1.48088700
H	-9.46287900	-1.06232100	1.94332800
H	-9.44160300	-2.20995000	-0.39089900
H	-10.91431800	-1.32398100	-0.81354900
H	-9.47753000	-1.21150100	-1.85179600
H	-9.67773400	1.33933800	-1.67751500
H	-11.06261600	1.13389800	-0.59753200
H	-9.69084400	2.12318300	-0.08231900

138

B_PPPM_-3233.843255 Hartree

C	-7.67027600	0.00429600	-0.58823800
C	-6.95268500	-1.18963000	-0.68504300
C	-5.55584600	-1.21279800	-0.81078200
C	-6.95182100	1.20226700	-0.68925600
C	-5.56008100	1.23196200	-0.81901000
C	-4.83750500	0.00935700	-0.78896800
C	-3.40237900	0.01524800	-0.58631800
C	-2.72723200	-1.22436000	-0.40213300
C	-3.39552000	-2.43643000	-0.88905600
C	-4.80589900	-2.44995400	-1.03064200
C	-5.43524500	-3.63369800	-1.46721000
C	-4.70145400	-4.76470900	-1.78690800
C	-3.30210200	-4.72754000	-1.71709200
C	-2.66562100	-3.57672000	-1.28565800
C	-0.72278900	0.01698700	0.23184300
C	-1.42727300	-1.21798400	0.15867600
C	-0.75885400	-2.36621000	0.77974700
C	-1.51261900	-3.38174000	1.50949900
C	-2.85392700	-3.18146500	1.91393900
C	-3.59226900	-4.18939800	2.50137800
C	-3.00488100	-5.44586100	2.71770200
C	-1.66862100	-5.63673200	2.41981200
C	-0.88088800	-4.60761400	1.85345800
C	0.57025000	-4.71156200	1.74734500
C	1.26645800	-5.87711600	2.14143900
C	2.64228300	-5.89032000	2.27554700
C	3.36785900	-4.70904600	2.06130800
C	2.71522800	-3.57214300	1.62464100
C	1.32201900	-3.55913900	1.37707400
C	0.63528500	-2.41843700	0.76658800
C	1.36522800	-1.26433600	0.24668200
C	2.67579700	-1.26007100	-0.30033100

C	3.22484000	-2.34405900	-1.10777800
C	4.60478100	-2.34725600	-1.44633000
C	5.09403500	-3.39904100	-2.25148200
C	4.26563000	-4.40412800	-2.72058500
C	2.89631600	-4.37440900	-2.42127900
C	2.39068400	-3.35474200	-1.63717900
C	-9.81738600	-1.35871600	-0.30526000
C	-9.19544900	0.04714600	-0.39587400
C	-9.84434800	0.78405700	-1.59089600
C	-9.52196800	0.80354000	0.91312300
C	7.66944800	-0.17285900	-0.89590200
C	7.07993700	0.90534400	-0.22787900
C	5.72104900	0.93532300	0.10115100
C	6.84799200	-1.24326900	-1.24773100
C	5.46776900	-1.25263700	-0.98975400
C	4.88993900	-0.14494100	-0.31271800
C	3.46829300	-0.10323700	-0.06065100
C	2.87774100	1.08262700	0.44862200
C	3.73955400	2.04071500	1.13226300
C	5.14477600	1.99501200	0.93299300
C	5.95189500	2.93417700	1.61086400
C	5.40886300	3.86650500	2.47842000
C	4.02941700	3.86114800	2.73106100
C	3.21743400	2.95566400	2.07482000
C	0.71110600	-0.00004100	0.32598500
C	1.45868100	1.21313200	0.30740400
C	0.73236400	2.46868400	0.25291400
C	1.32677500	3.76408500	-0.09378000
C	2.47796900	3.85238500	-0.90850700
C	2.99238800	5.07536000	-1.30027200
C	2.36463400	6.26259100	-0.89514000
C	1.20979900	6.20110000	-0.13387200
C	0.66273500	4.96617000	0.27235400
C	-0.57916600	4.87753900	1.03165900
C	-1.13223500	5.97345600	1.72439300
C	-2.33330700	5.86145400	2.40527800
C	-3.01100900	4.63258200	2.42755300
C	-2.48306100	3.54053400	1.76283300
C	-1.26986900	3.63723900	1.04510900
C	-0.65574700	2.45916600	0.43977400
C	-1.40106100	1.25709000	0.09537600
C	-2.72443700	1.25530400	-0.43473100
C	-3.39610000	2.44434900	-0.97940000
C	-4.81126600	2.46153300	-1.08070400
C	-5.44905800	3.62573500	-1.55661200
C	-4.72131200	4.72713000	-1.97638400
C	-3.32123800	4.66950900	-1.97996100
C	-2.67716700	3.54247400	-1.49997300
C	9.97210100	-0.00861900	0.11089800
C	9.17553100	-0.14147300	-1.20809200
C	9.48149500	1.07087400	-2.11838600
C	9.65484000	-1.41531000	-1.92866600
H	-7.48005700	-2.13275500	-0.63621800
H	-7.49127800	2.14165400	-0.64670300
H	-6.51165900	-3.65070800	-1.60125700
H	-5.21002400	-5.66254500	-2.12739200
H	-2.71417200	-5.59190300	-2.01254900
H	-1.58297300	-3.54848900	-1.25577200

H	-3.31358400	-2.21389700	1.76015500
H	-4.62229600	-4.00540800	2.79317500
H	-3.58300400	-6.25393100	3.15764500
H	-1.21569500	-6.58999100	2.66645300
H	0.71501100	-6.78173700	2.37094900
H	3.14967300	-6.80060600	2.58323300
H	4.44051700	-4.68261100	2.23080200
H	3.29070800	-2.67013300	1.47200800
H	6.13989800	-3.42254700	-2.53495600
H	4.67555400	-5.19836200	-3.33851900
H	2.23053400	-5.13843800	-2.81265400
H	1.32758300	-3.32102400	-1.43244000
H	-9.41430700	-1.92684900	0.54079400
H	-10.90042100	-1.27476200	-0.16203100
H	-9.64949900	-1.93783900	-1.22053400
H	-9.62417100	0.26813300	-2.53229500
H	-10.93359200	0.82359700	-1.46794600
H	-9.48194200	1.81326900	-1.68076100
H	-9.14448500	1.83118900	0.89451200
H	-10.60705100	0.84857200	1.06688900
H	-9.07430800	0.29828500	1.77640900
H	7.70509900	1.74507500	0.04960700
H	7.28894400	-2.09406800	-1.74614700
H	7.02855000	2.91067100	1.48490400
H	6.05746300	4.57384100	2.98797900
H	3.59810800	4.55345800	3.44840900
H	2.15760800	2.93494400	2.29723100
H	2.95300000	2.94251100	-1.25337700
H	3.87404300	5.10972000	-1.93408200
H	2.76241600	7.22595300	-1.20230900
H	0.69610400	7.12191600	0.12238900
H	-0.59675200	6.91704900	1.74802000
H	-2.73756600	6.71880400	2.93648900
H	-3.94125900	4.53045300	2.97933400
H	-2.99624100	2.58690100	1.80637000
H	-6.52973000	3.64390100	-1.64982200
H	-5.23626600	5.60928100	-2.34690300
H	-2.73644500	5.49782100	-2.36957100
H	-1.59611000	3.49900800	-1.54205600
H	9.76911400	-0.85647300	0.77482600
H	11.04910100	0.01533200	-0.09528300
H	9.71656500	0.90814800	0.65253100
H	9.20368300	2.01763900	-1.64350800
H	10.55319300	1.11441000	-2.34788500
H	8.93155500	0.99661200	-3.06336600
H	9.14824200	-1.55424200	-2.89059100
H	10.72965600	-1.34353300	-2.12899000
H	9.49148800	-2.31190200	-1.31979800

138
C_MPPM_-3233.826687 Hartree

C	-7.69591300	-0.04609400	0.29893800
C	-7.00822900	-1.23817100	0.05862600
C	-5.66280800	-1.25314000	-0.33764600
C	-7.01718700	1.15716100	0.05901300
C	-5.67714500	1.19130000	-0.33587800
C	-4.95952000	-0.02852400	-0.43926600
C	-3.51195600	-0.01995800	-0.49665300
C	-2.80812000	-1.24975000	-0.46919300

C	-3.55652600	-2.42713100	-0.93197400
C	-4.96531800	-2.46634200	-0.76535500
C	-5.66700700	-3.62571100	-1.15410800
C	-5.01722500	-4.69458000	-1.75046300
C	-3.64543600	-4.60600800	-2.02399900
C	-2.93549600	-3.48542900	-1.62851000
C	-0.71887500	-0.00406600	-0.07673800
C	-1.42502400	-1.24738500	-0.12481700
C	-0.67369400	-2.46747400	0.11615900
C	-1.23415200	-3.70695300	0.65520700
C	-2.35743300	-3.68988100	1.51018900
C	-2.83000100	-4.85190200	2.09384800
C	-2.18413100	-6.07336300	1.84820900
C	-1.06034800	-6.10736000	1.03849000
C	-0.56144700	-4.93722500	0.43228500
C	0.61683100	-4.93139600	-0.42752800
C	1.12865000	-6.09691700	-1.03180000
C	2.25211700	-6.05187300	-1.84144000
C	2.88458500	-4.82379700	-2.08890500
C	2.39911700	-3.66608600	-1.50724600
C	1.27592300	-3.69407800	-0.65244900
C	0.70188100	-2.46003300	-0.11527700
C	1.43957500	-1.23145300	0.12464800
C	2.82225000	-1.21796300	0.46855500
C	3.58391000	-2.38723500	0.93273000
C	4.99253800	-2.41178300	0.76626900
C	5.70743300	-3.56121200	1.15882500
C	5.06961700	-4.63631000	1.75737800
C	3.69695100	-4.56245900	2.02981800
C	2.97458600	-3.45051000	1.63174800
C	-9.73948500	-1.41924700	0.99637800
C	-9.15572700	-0.01060300	0.78186500
C	-10.02795300	0.71769600	-0.26744700
C	-9.22847700	0.74934000	2.12699700
C	7.69589700	0.04612800	-0.29909400
C	7.00823400	1.23819600	-0.05867100
C	5.66283300	1.25314700	0.33766800
C	7.01718500	-1.15713900	-0.05918000
C	5.67716700	-1.19129200	0.33578800
C	4.95954800	0.02852900	0.43925400
C	3.51198400	0.01996200	0.49666400
C	2.80814800	1.24975300	0.46924100
C	3.55656000	2.42711200	0.93207500
C	4.96535200	2.46632500	0.76545700
C	5.66705000	3.62566400	1.15427800
C	5.01727500	4.69450600	1.75068900
C	3.64548300	4.60593200	2.02421000
C	2.93553400	3.48538000	1.62865800
C	0.71890500	0.00407800	0.07675800
C	1.42505300	1.24739800	0.12485400
C	0.67371500	2.46749100	-0.11608600
C	1.23415100	3.70700000	-0.65509600
C	2.35740300	3.68997800	-1.51011700
C	2.82994600	4.85203000	-2.09373500
C	2.18407700	6.07347500	-1.84801600
C	1.06031600	6.10742400	-1.03826600
C	0.56143900	4.93725600	-0.43210400
C	-0.61683300	4.93137700	0.42771600

C	-1.12866500	6.09686300	1.03204600
C	-2.25214700	6.05176900	1.84166200
C	-2.88462000	4.82367900	2.08904200
C	-2.39913600	3.66600000	1.50733000
C	-1.27592200	3.69404500	0.65256400
C	-0.70186200	2.46003200	0.11534000
C	-1.43954900	1.23145900	-0.12463700
C	-2.82222000	1.21796700	-0.46857300
C	-3.58387100	2.38722400	-0.93280600
C	-4.99250300	2.41177700	-0.76637600
C	-5.70739000	3.56119800	-1.15897500
C	-5.06956700	4.63627600	-1.75755500
C	-3.69689900	4.56240800	-2.02998300
C	-2.97454300	3.45047200	-1.63186600
C	10.02796700	-0.71788500	0.26689700
C	9.15565700	0.01065200	-0.78217700
C	9.73946500	1.41930400	-0.99650200
C	9.22820300	-0.74905500	-2.12745500
H	-7.51472300	-2.18443600	0.19529300
H	-7.54329700	2.09516600	0.19744100
H	-6.74532900	-3.66242500	-1.03881400
H	-5.58101800	-5.57383400	-2.05001000
H	-3.13658000	-5.40670600	-2.55296600
H	-1.88108200	-3.41613200	-1.86769300
H	-2.84743500	-2.74585600	1.71931700
H	-3.69326700	-4.81199600	2.75216000
H	-2.54799600	-6.98711100	2.31003000
H	-0.53843300	-7.04793400	0.89314900
H	0.61710400	-7.04294600	-0.88500000
H	2.62604400	-6.96230400	-2.30177400
H	3.74747100	-4.77543000	-2.74714700
H	2.87870300	-2.71706900	-1.71792700
H	6.78630200	-3.58505200	1.04473600
H	5.64323400	-5.50828300	2.05951000
H	3.19673200	-5.36764700	2.56020700
H	1.91935300	-3.39270700	1.87044300
H	-9.17414300	-1.98243700	1.74756900
H	-10.77355700	-1.33991900	1.34998600
H	-9.75179700	-2.00017500	0.06716600
H	-9.99368200	0.19730700	-1.23126400
H	-11.07250300	0.75502500	0.06519500
H	-9.69380200	1.74745600	-0.43167300
H	-8.86813700	1.77893900	2.03315700
H	-10.26386800	0.78902800	2.48725100
H	-8.61969900	0.25005700	2.88927000
H	7.51472600	2.18446700	-0.19530900
H	7.54328100	-2.09514000	-0.19768600
H	6.74537300	3.66237100	1.03899100
H	5.58107400	5.57373700	2.05029100
H	3.13663000	5.40661000	2.55321100
H	1.88111600	3.41608500	1.86782500
H	2.84740400	2.74596700	-1.71931100
H	3.69319100	4.81216000	-2.75207600
H	2.54792100	6.98724800	-2.30980500
H	0.53839400	7.04798600	-0.89287000
H	-0.61712100	7.04290100	0.88530500
H	-2.62608400	6.96217300	2.30204200
H	-3.74752300	4.77527400	2.74725900

H	-2.87872900	2.71697000	1.71794000
H	-6.78626000	3.58504600	-1.04490500
H	-5.64317900	5.50823900	-2.05972300
H	-3.19667300	5.36757100	-2.56040300
H	-1.91931600	3.39265200	-1.87057300
H	9.69374800	-1.74764000	0.43102000
H	11.07246400	-0.75526100	-0.06590700
H	9.99389100	-0.19763800	1.23079800
H	9.75195000	2.00005000	-0.06717900
H	10.77347800	1.33998700	-1.35028200
H	9.17404000	1.98267300	-1.74749700
H	8.61935900	-0.24959900	-2.88956400
H	10.26354800	-0.78873800	-2.48784200
H	8.86781200	-1.77864900	-2.03375600

138

D_PMPM_-3233.845834 Hartree

C	7.50221300	0.00626700	-1.23811600
C	6.78000900	1.19978300	-1.22140100
C	5.38061900	1.22683500	-1.12259200
C	6.77435500	-1.18956500	-1.21096600
C	5.38022700	-1.21820000	-1.11474600
C	4.66944500	0.00669300	-0.98278500
C	3.27737200	0.00783800	-0.58140800
C	2.63086100	1.25320000	-0.32378500
C	3.23789900	2.46492000	-0.88773800
C	4.61551800	2.46947700	-1.22422300
C	5.18690800	3.65300300	-1.73710100
C	4.42624800	4.79149100	-1.94598500
C	3.05032000	4.76407300	-1.68222600
C	2.47144400	3.61539700	-1.17201700
C	0.72258900	0.01065700	0.52282700
C	1.39002600	1.24884900	0.35870000
C	0.69643600	2.38655900	0.98829800
C	1.42385200	3.41560700	1.72274700
C	2.80835700	3.30898600	1.99736600
C	3.51176600	4.33729500	2.59219500
C	2.84717800	5.52225100	2.94534800
C	1.48006300	5.61865200	2.76644400
C	0.72901100	4.56434900	2.19620800
C	-0.72905400	4.56439700	2.19604600
C	-1.48017800	5.61876800	2.76606100
C	-2.84733800	5.52244400	2.94467100
C	-3.51191100	4.33750500	2.59143100
C	-2.80842600	3.30913400	1.99679900
C	-1.42385600	3.41567500	1.72248800
C	-0.69635100	2.38657700	0.98821000
C	-1.38991000	1.24885700	0.35860600
C	-2.63073200	1.25320800	-0.32390200
C	-3.23775300	2.46493200	-0.88785800
C	-4.61536700	2.46950100	-1.22436200
C	-5.18673000	3.65302600	-1.73727100
C	-4.42605100	4.79150300	-1.94615400
C	-3.05013100	4.76407700	-1.68235300
C	-2.47127800	3.61540100	-1.17211700
C	9.66793400	1.36573800	-1.33993800
C	9.03821900	-0.03904500	-1.29950400
C	9.47979700	-0.80016700	-2.57149800
C	9.57641000	-0.77200700	-0.04854500

C	-7.50213600	0.00637500	-1.23809100
C	-6.77431900	-1.18947900	-1.21084800
C	-5.38019100	-1.21815900	-1.11465200
C	-6.77989000	1.19986800	-1.22147600
C	-5.38049800	1.22688000	-1.12268700
C	-4.66936000	0.00672200	-0.98281600
C	-3.27727700	0.00785300	-0.58147500
C	-2.62971800	-1.23634700	-0.31900400
C	-3.23500400	-2.45158700	-0.87921300
C	-4.61231200	-2.46023800	-1.21403200
C	-5.18173900	-3.64474000	-1.72540200
C	-4.41896000	-4.78236200	-1.93299400
C	-3.04316300	-4.75150900	-1.67011100
C	-2.46617000	-3.60047000	-1.16242500
C	-0.72248900	0.01066500	0.52279100
C	-1.38976100	-1.22880900	0.36308300
C	-0.69622600	-2.36445300	0.99686800
C	-1.42388800	-3.39141500	1.73381300
C	-2.80855700	-3.28431100	2.00744500
C	-3.51214000	-4.31140300	2.60415800
C	-2.84756300	-5.49551800	2.96022800
C	-1.48034000	-5.59218000	2.78228200
C	-0.72910700	-4.53904400	2.21013300
C	0.72893400	-4.53903300	2.21027200
C	1.48005600	-5.59220000	2.78251100
C	2.84723300	-5.49551900	2.96078900
C	3.51185500	-4.31135200	2.60498000
C	2.80839300	-3.28425200	2.00813800
C	1.42380200	-3.39138300	1.73411400
C	0.69628200	-2.36444800	0.99696500
C	1.38985500	-1.22882900	0.36315000
C	2.62978400	-1.23636400	-0.31900600
C	3.23498400	-2.45154700	-0.87944700
C	4.61228800	-2.46022700	-1.21428200
C	5.18165000	-3.64467900	-1.72583800
C	4.41880500	-4.78222000	-1.93362600
C	3.04300500	-4.75131700	-1.67076900
C	2.46608100	-3.60032800	-1.16289300
C	-9.57637100	-0.77140400	-0.04824700
C	-9.03814400	-0.03888900	-1.29945300
C	-9.47975300	-0.80042200	-2.57118900
C	-9.66779700	1.36590800	-1.34036000
H	7.31134900	2.14075600	-1.26148600
H	7.31319200	-2.12928800	-1.24395400
H	6.23378400	3.66484100	-2.02054400
H	4.89051800	5.68724100	-2.34945500
H	2.43314500	5.63374500	-1.88923000
H	1.40308400	3.59581100	-0.99712700
H	3.33112700	2.39917900	1.73485800
H	4.57555700	4.22536800	2.78162000
H	3.39544600	6.34811400	3.39042700
H	0.97780600	6.51889000	3.10129200
H	-0.97795100	6.51901000	3.10094800
H	-3.39566300	6.34836200	3.38957700
H	-4.57575000	4.22564400	2.78062500
H	-3.33118400	2.39934500	1.73419200
H	-6.23360100	3.66487300	-2.02073100
H	-4.89030200	5.68725200	-2.34964500

H	-2.43294400	5.63374200	-1.88934600
H	-1.40292300	3.59580400	-0.99719400
H	9.41487000	1.95100300	-0.44853100
H	10.75941900	1.27889200	-1.37964100
H	9.34841100	1.92893300	-2.22425300
H	9.10775600	-0.30050700	-3.47302500
H	10.57423100	-0.84237800	-2.62969100
H	9.10646900	-1.82947000	-2.58159400
H	9.19736700	-1.79711200	0.01706800
H	10.67181000	-0.82159300	-0.07707900
H	9.28144000	-0.24686800	0.86697100
H	-7.31319200	-2.12918600	-1.24373600
H	-7.31119800	2.14085700	-1.26160300
H	-6.22863300	-3.65811700	-2.00895300
H	-4.88158500	-5.67955300	-2.33511900
H	-2.42422600	-5.62017600	-1.87607300
H	-1.39769400	-3.57829100	-0.98842800
H	-3.33124800	-2.37510500	1.74263900
H	-4.57603900	-4.19920400	2.79281100
H	-3.39598500	-6.32049000	3.40676700
H	-0.97816600	-6.49166000	3.11930000
H	0.97782500	-6.49172300	3.11932700
H	3.39557300	-6.32051000	3.40739500
H	4.57570000	-4.19912300	2.79392100
H	3.33112900	-2.37501500	1.74354900
H	6.22854300	-3.65806900	-2.00939400
H	4.88137800	-5.67937100	-2.33590000
H	2.42401300	-5.61990600	-1.87689700
H	1.39760300	-3.57811300	-0.98892500
H	-9.28132600	-0.24600300	0.86709500
H	-10.67177600	-0.82089100	-0.07673500
H	-9.19743000	-1.79652500	0.01768300
H	-9.10639900	-1.82972000	-2.58097000
H	-10.57418900	-0.84267600	-2.62933100
H	-9.10775300	-0.30104100	-3.47288800
H	-9.34847900	1.92868600	-2.22501300
H	-10.75929700	1.27910200	-1.37974100
H	-9.41446600	1.95156400	-0.44928400

138
E_PPMM_-3233.843602 Hartree

C	7.77674500	-0.00132400	-0.03422200
C	7.05587300	1.07436300	-0.55079100
C	5.65218400	1.10142000	-0.56841700
C	7.05259200	-1.07248000	0.50100200
C	5.65481600	-1.08964800	0.54938000
C	4.93807500	0.00973500	-0.00436000
C	3.49418400	0.01214200	0.00385300
C	2.78980100	1.18812200	-0.36531700
C	3.49130000	2.22662300	-1.10592400
C	4.90812400	2.19293200	-1.20390700
C	5.55090700	3.20515300	-1.94984100
C	4.83586300	4.20674100	-2.58384100
C	3.43454500	4.21343500	-2.51849000
C	2.78018900	3.23168500	-1.79968100
C	0.71345900	0.01180600	0.00636700
C	1.41312800	1.25249200	0.00741800
C	0.70122900	2.44283100	0.45078700
C	1.41313200	3.60487800	0.99328100

C	2.76082300	3.55027000	1.41833600
C	3.46223000	4.68962100	1.76449800
C	2.82957700	5.94013200	1.70616000
C	1.47929700	6.00656300	1.41429600
C	0.72968500	4.84664200	1.11427800
C	-0.72998200	4.84662000	1.11414300
C	-1.47969900	6.00651300	1.41400700
C	-2.83003600	5.94003100	1.70560100
C	-3.46266100	4.68949900	1.76380700
C	-2.76114100	3.55017000	1.41779900
C	-1.41335900	3.60483100	0.99304700
C	-0.70131800	2.44280300	0.45071300
C	-1.41315200	1.25243400	0.00735100
C	-2.78982700	1.18803600	-0.36535500
C	-3.49136900	2.22656800	-1.10587300
C	-4.90819300	2.19283700	-1.20384400
C	-5.55101400	3.20507400	-1.94972300
C	-4.83600500	4.20672400	-2.58366800
C	-3.43468800	4.21347200	-2.51831000
C	-2.78029400	3.23170300	-1.79956000
C	9.94433600	1.20964600	-0.65717400
C	9.31462300	-0.05002300	-0.03438800
C	9.78582000	-1.27653400	-0.85019800
C	9.82430200	-0.17220600	1.42065200
C	-7.77673700	-0.00152500	-0.03417100
C	-7.05254700	-1.07267900	0.50100900
C	-5.65476900	-1.08981900	0.54935300
C	-7.05589900	1.07419000	-0.55072800
C	-5.65221200	1.10127600	-0.56839000
C	-4.93806500	0.00958900	-0.00438300
C	-3.49417200	0.01202700	0.00379900
C	-2.79026300	-1.16414600	0.37603300
C	-3.49482500	-2.20278200	1.11517700
C	-4.91190000	-2.17401200	1.20068500
C	-5.55799100	-3.18162100	1.94863800
C	-4.84514600	-4.17752500	2.59477600
C	-3.44367000	-4.18126500	2.53917400
C	-2.78619500	-3.20158500	1.81966400
C	-0.71344300	0.01177400	0.00633700
C	-1.41382000	-1.22931000	0.00685700
C	-0.70107500	-2.42140000	-0.43233000
C	-1.41278200	-3.58466000	-0.97227400
C	-2.75977100	-3.53094600	-1.39980400
C	-3.46124800	-4.67121500	-1.74268700
C	-2.82934300	-5.92187100	-1.67848100
C	-1.47943800	-5.98787200	-1.38483800
C	-0.72963800	-4.82710700	-1.08846300
C	0.72992100	-4.82707800	-1.08839400
C	1.47980000	-5.98781100	-1.38469900
C	2.82973400	-5.92175200	-1.67819900
C	3.46159800	-4.67107000	-1.74232500
C	2.76003700	-3.53083100	-1.39951600
C	1.41300100	-3.58460400	-0.97214500
C	0.70119500	-2.42137100	-0.43228100
C	1.41387700	-1.22925300	0.00691900
C	2.79031200	-1.16404600	0.37610600
C	3.49491000	-2.20266600	1.11523700
C	4.91198400	-2.17385300	1.20073400

C	5.55811000	-3.18144000	1.94868600
C	4.84530000	-4.17736500	2.59483000
C	3.44382300	-4.18115200	2.53923000
C	2.78631300	-3.20149400	1.81972200
C	-9.82423700	-0.17251000	1.42076800
C	-9.31461400	-0.05025000	-0.03428500
C	-9.78582100	-1.27672900	-0.85013600
C	-9.94437000	1.20943800	-0.65698900
H	7.59574100	1.91684800	-0.95834000
H	7.60190200	-1.91864900	0.89514600
H	6.62969100	3.19621800	-2.05660400
H	5.36187400	4.97013000	-3.15063000
H	2.86328400	4.97627100	-3.03982800
H	1.69720600	3.22376000	-1.76858700
H	3.26007200	2.59280100	1.47719600
H	4.49991400	4.61033900	2.07561700
H	3.38037700	6.84742900	1.93892500
H	0.98585600	6.97078800	1.45918900
H	-0.98630500	6.97075900	1.45898300
H	-3.38091800	6.84731000	1.93824300
H	-4.50041100	4.61018400	2.07470000
H	-3.26037000	2.59267900	1.47652800
H	-6.62979700	3.19610600	-2.05648400
H	-5.36204500	4.97012500	-3.15041400
H	-2.86345600	4.97636200	-3.03960100
H	-1.69731000	3.22381500	-1.76846400
H	9.67087600	2.11602700	-0.10495300
H	11.03652600	1.12570800	-0.63401100
H	9.64473900	1.33905300	-1.70342800
H	9.43715500	-1.21093200	-1.88707000
H	10.88126800	-1.33027700	-0.85982500
H	9.41013000	-2.21526100	-0.42994600
H	9.44899200	-1.07747900	1.90869500
H	10.92024800	-0.21273800	1.43933900
H	9.50322400	0.68804800	2.01883000
H	-7.60183000	-1.91886500	0.89515500
H	-7.59579400	1.91668100	-0.95823000
H	-6.63757500	-3.17263600	2.04814500
H	-5.37336600	-4.93806500	3.16331500
H	-2.87432800	-4.93961200	3.06909000
H	-1.70296800	-3.19076200	1.79651800
H	-3.25820500	-2.57335600	-1.46353600
H	-4.49835800	-4.59255500	-2.05587700
H	-3.38033100	-6.82976200	-1.90847000
H	-0.98629000	-6.95243700	-1.42569600
H	0.98669600	-6.95239600	-1.42561200
H	3.38078300	-6.82961900	-1.90813200
H	4.49874000	-4.59236900	-2.05539600
H	3.25844000	-2.57321900	-1.46317400
H	6.63769400	-3.17242100	2.04818700
H	5.37354500	-4.93788700	3.16336800
H	2.87450800	-4.93951800	3.06914800
H	1.70308600	-3.19070500	1.79658100
H	-9.50315000	0.68772100	2.01897500
H	-10.92018200	-0.21306000	1.43949400
H	-9.44889300	-1.07779900	1.90875500
H	-9.41010200	-2.21547100	-0.42994300
H	-10.88126900	-1.33048900	-0.85972500

H -9.43719500 -1.21107300 -1.88701700
H -9.64480800 1.33890200 -1.70324600
H -11.03655700 1.12547800 -0.63379400
H -9.67090900 2.11579700 -0.10473200
138
TS1a_-3233.786784 Hartree_Imaginary frequency: -22.88 cm-1
C -7.28929300 0.06197800 1.16775800
C -6.72714000 -1.13158700 0.70249000
C -5.56057100 -1.14338500 -0.07300700
C -6.68768500 1.26545900 0.77004100
C -5.51404300 1.29589700 0.01183200
C -4.88523200 0.07261000 -0.32789500
C -3.50189700 0.05699100 -0.76999200
C -2.85883400 -1.17917600 -1.03978400
C -3.81338000 -2.22152900 -1.46232300
C -5.08824800 -2.30080100 -0.83644600
C -5.96853600 -3.33224600 -1.21181900
C -5.67693800 -4.17072600 -2.28053500
C -4.53023600 -3.93371200 -3.04428500
C -3.62303500 -2.96515400 -2.63563800
C -0.72640600 -0.03170500 -0.62418000
C -1.43603300 -1.27508900 -0.86891600
C -0.72929800 -2.51600900 -0.48580400
C -1.11548700 -3.93357700 -0.64391800
C -1.40046100 -4.48464300 -1.90659900
C -1.68153800 -5.83170700 -2.07689100
C -1.66278500 -6.69140000 -0.97313100
C -1.23384700 -6.20526300 0.25229800
C -0.89039300 -4.84965400 0.42359300
C -0.11114600 -4.40481700 1.57224000
C -0.03954600 -5.10816700 2.78761300
C 0.85732900 -4.73286400 3.77688400
C 1.70672600 -3.63672800 3.56758200
C 1.63281400 -2.91370400 2.38770600
C 0.71673500 -3.26902100 1.37370000
C 0.46227000 -2.38585300 0.23524500
C 1.32868800 -1.27674900 -0.08104000
C 2.74878800 -1.36701600 -0.14096600
C 3.46898600 -2.61170800 -0.41688100
C 4.88851000 -2.64521300 -0.31763100
C 5.55249000 -3.87133600 -0.54785500
C 4.87020400 -5.01504700 -0.92120700
C 3.48408100 -4.95702200 -1.11785200
C 2.80745200 -3.77678300 -0.87570100
C -9.07138100 -1.30762800 2.39043600
C -8.54453000 0.10019500 2.05525000
C -9.66839900 0.87321700 1.32624800
C -8.20605500 0.81579100 3.38398900
C 7.76243800 -0.21603300 0.18312900
C 7.04258900 0.96099800 0.41741400
C 5.64664500 0.99288800 0.44879600
C 7.03610700 -1.38815600 -0.01251400
C 5.63038400 -1.41407700 -0.03268400
C 4.92095400 -0.20038300 0.16430400
C 3.48118600 -0.16516400 0.04343700
C 2.80366200 1.08463600 0.10996100
C 3.49259300 2.19832800 0.76741000
C 4.90678600 2.18406900 0.87438000

C	5.55105900	3.27597100	1.49508600
C	4.83407400	4.32824000	2.03903400
C	3.43308000	4.29111900	2.02477200
C	2.78168200	3.23734300	1.41058700
C	0.69372900	-0.05135400	-0.38242000
C	1.46503100	1.14935000	-0.37193400
C	0.78176800	2.39476500	-0.64305600
C	1.42635300	3.60574100	-1.13834800
C	2.60060000	3.54153000	-1.91763000
C	3.14416600	4.68266900	-2.48157300
C	2.52307700	5.92600000	-2.28523200
C	1.36634100	6.00922600	-1.52635800
C	0.80043400	4.86348100	-0.93356100
C	-0.37136700	4.90770100	-0.06575300
C	-0.81275500	6.09685000	0.55063500
C	-1.88420900	6.09785800	1.42810100
C	-2.52802700	4.89076000	1.74252200
C	-2.12025500	3.71374100	1.14238600
C	-1.06797700	3.69767400	0.19957500
C	-0.58741200	2.44193700	-0.37796100
C	-1.38679500	1.23909400	-0.58973300
C	-2.79266700	1.27871500	-0.77932600
C	-3.60419300	2.47620400	-1.03560000
C	-4.93687400	2.51696600	-0.55395200
C	-5.69543400	3.68766200	-0.74254400
C	-5.17744000	4.77248200	-1.43514400
C	-3.89603200	4.69223500	-1.99714900
C	-3.12711700	3.55610900	-1.80316000
C	9.82231200	0.33585300	1.52175400
C	9.29975800	-0.17333000	0.15787300
C	9.76404400	0.78744900	-0.96161700
C	9.92424700	-1.55569900	-0.10844100
H	-7.19694900	-2.07793400	0.93957500
H	-7.13377500	2.20632300	1.07422800
H	-6.93598500	-3.41059900	-0.72605300
H	-6.38211900	-4.94446100	-2.57143200
H	-4.35035900	-4.49402600	-3.95735300
H	-2.77470400	-2.72263500	-3.26145500
H	-1.32608600	-3.84869600	-2.77515300
H	-1.89578900	-6.21494600	-3.07059000
H	-1.90086500	-7.74511400	-1.08926000
H	-1.07522700	-6.89907800	1.07155500
H	-0.70101700	-5.95239200	2.95681500
H	0.89771500	-5.28576000	4.71141400
H	2.41087200	-3.33622500	4.33844500
H	2.26299000	-2.04232500	2.25549300
H	6.62882100	-3.93145600	-0.43867700
H	5.41339500	-5.94154900	-1.08632300
H	2.93598400	-5.82910000	-1.46306300
H	1.74368400	-3.74858900	-1.06671900
H	-8.32319500	-1.90446200	2.92427000
H	-9.95347700	-1.22731900	3.03544400
H	-9.37034700	-1.85524900	1.48936600
H	-9.92061100	0.38867900	0.37625600
H	-10.57294400	0.90470600	1.94582800
H	-9.37849400	1.90628800	1.10830400
H	-7.86305700	1.84216600	3.21749000
H	-9.09178900	0.85975200	4.02959400

H	-7.41533500	0.28197900	3.92313700
H	7.59159800	1.87996800	0.58323300
H	7.57522800	-2.30752500	-0.18448000
H	6.63083700	3.27919900	1.59412100
H	5.35796800	5.15320100	2.51412600
H	2.85410600	5.07553900	2.50382500
H	1.69990600	3.20032300	1.44429700
H	3.06854800	2.57812900	-2.08733500
H	4.04325900	4.61017800	-3.08704400
H	2.93983000	6.82186400	-2.73738800
H	0.87917200	6.97152200	-1.40429700
H	-0.27782200	7.02293900	0.36596000
H	-2.20111000	7.02548400	1.89679200
H	-3.33918800	4.87394400	2.46483400
H	-2.60564500	2.78147300	1.40553200
H	-6.72077400	3.72312100	-0.38768700
H	-5.78274200	5.66345600	-1.57794900
H	-3.50351400	5.51472700	-2.58805900
H	-2.13887900	3.49481800	-2.24488300
H	9.50443800	-0.32858300	2.33314000
H	10.91848700	0.37437100	1.51936800
H	9.45403900	1.34148400	1.74955500
H	9.39088100	1.80501200	-0.80621200
H	10.85948200	0.83464500	-0.99343700
H	9.40728500	0.44579700	-1.93981200
H	9.61387000	-1.96177400	-1.07795000
H	11.01664600	-1.47106700	-0.11981300
H	9.65726400	-2.27960400	0.66997100

138

TS1b_-3233.777849 Hartree_Imaginary frequency: -24.88 cm-1

C	6.56529700	0.18750100	-2.56282700
C	6.37779300	1.00150800	-1.44227200
C	5.41615500	0.70822200	-0.47123000
C	5.78498600	-0.96746600	-2.65808900
C	4.78931500	-1.29321000	-1.72353500
C	4.54593300	-0.40158800	-0.64984900
C	3.37027100	-0.55855800	0.18223700
C	3.07791000	0.46467500	1.14341700
C	4.28191800	1.11105400	1.69118300
C	5.38539600	1.36536900	0.83490100
C	6.51990000	2.01748100	1.35578700
C	6.63436400	2.28761000	2.71216300
C	5.64177800	1.83242000	3.58579200
C	4.49315400	1.24682600	3.07353100
C	0.78781100	-0.31648800	1.05156000
C	1.69022700	0.74822500	1.37528300
C	1.04952500	2.04796800	1.66100900
C	1.64691600	3.13009000	2.44375400
C	2.30729100	2.89983000	3.66105200
C	2.92981200	3.92056700	4.36096800
C	2.90341800	5.22255300	3.84615200
C	2.11816800	5.50113000	2.73784800
C	1.40337100	4.48166800	2.07456700
C	0.21067200	4.76905100	1.28383700
C	-0.18553300	6.08245400	0.95693200
C	-1.49525500	6.36972800	0.61017400
C	-2.45651900	5.35005100	0.65285900
C	-2.06535700	4.04406900	0.89486500

C	-0.71072100	3.70106600	1.09535000
C	-0.20670700	2.31645800	1.08977000
C	-1.00774500	1.22646000	0.55865100
C	-2.16369400	1.32247300	-0.27141200
C	-2.33343600	2.26620200	-1.36579100
C	-3.61632000	2.44598400	-1.94927400
C	-3.73844900	3.34789300	-3.02804600
C	-2.64584300	4.04050300	-3.52323800
C	-1.37432600	3.83012100	-2.96936200
C	-1.22549100	2.94864200	-1.91536300
C	8.36660200	1.80850300	-3.38287800
C	7.58804100	0.51033000	-3.66547000
C	6.84817600	0.67720500	-5.01336700
C	8.60843200	-0.64627800	-3.77868800
C	-7.13930300	1.09723300	-1.49965900
C	-6.88072900	0.05597400	-0.60109400
C	-5.60529100	-0.19303700	-0.08272400
C	-6.06527100	1.89426200	-1.89686300
C	-4.75515500	1.67092200	-1.44564800
C	-4.52117600	0.61978300	-0.51848700
C	-3.18774000	0.36979400	-0.02755600
C	-2.91378300	-0.81912000	0.69466000
C	-4.02482800	-1.51735000	1.32327200
C	-5.35998500	-1.21698100	0.93794700
C	-6.41339300	-1.89139100	1.59119300
C	-6.17368900	-2.81612200	2.59414100
C	-4.85769200	-3.07705100	3.00297300
C	-3.80728600	-2.42906400	2.38066500
C	-0.61910400	-0.10662200	0.87996100
C	-1.53407000	-1.19959700	0.77636600
C	-1.00965600	-2.55212900	0.79414600
C	-1.77455700	-3.76171700	0.46153200
C	-2.93325800	-3.71315500	-0.34758100
C	-3.62967400	-4.85718400	-0.69216400
C	-3.18287500	-6.10930200	-0.24709100
C	-2.01004600	-6.19319200	0.48241900
C	-1.27168400	-5.04133500	0.83060400
C	0.02997600	-5.13682300	1.48181200
C	0.49491400	-6.31722100	2.09666100
C	1.77846200	-6.40421800	2.61000600
C	2.63880600	-5.29858000	2.52982300
C	2.19636300	-4.11952100	1.95666600
C	0.88911400	-4.00697100	1.43091400
C	0.37231100	-2.72951800	0.95380400
C	1.25948800	-1.61031700	0.71368000
C	2.47907500	-1.64332600	-0.02770000
C	2.76697300	-2.62010800	-1.08934900
C	3.95015800	-2.48155300	-1.87094200
C	4.26324100	-3.47453900	-2.82495700
C	3.40852500	-4.52881600	-3.08958500
C	2.17744000	-4.58975400	-2.42521700
C	1.86990500	-3.65331800	-1.45512200
C	-9.50819800	1.60011600	-0.80968600
C	-8.57359600	1.32036900	-2.00992100
C	-9.05492500	0.05144700	-2.75155300
C	-8.67497200	2.51059000	-2.98194900
H	6.99013100	1.88396700	-1.31033400
H	5.92260700	-1.60936300	-3.51897400

H	7.36086000	2.23071700	0.70432100
H	7.52805700	2.77150700	3.09657800
H	5.77076300	1.92015600	4.66090300
H	3.76745300	0.81981900	3.75035300
H	2.26429300	1.90430600	4.07782800
H	3.43261500	3.70839200	5.30003100
H	3.42221300	6.02774800	4.35918400
H	1.97977700	6.53538100	2.44204600
H	0.53477100	6.89154800	1.02128100
H	-1.78321900	7.38851700	0.36558100
H	-3.50526600	5.57325200	0.47995800
H	-2.81941900	3.26960000	0.91973700
H	-4.70086500	3.49088700	-3.50668000
H	-2.77367900	4.72595700	-4.35663900
H	-0.50684500	4.34487100	-3.37241500
H	-0.24023300	2.76984000	-1.50048800
H	8.94114500	1.74328000	-2.45173200
H	9.07603600	1.99772700	-4.19617300
H	7.69999300	2.67576800	-3.31661200
H	6.11886300	1.49327200	-4.95853600
H	7.56162100	0.90822700	-5.81396300
H	6.30995200	-0.23282900	-5.29761100
H	8.12361000	-1.59520200	-4.03002700
H	9.34376800	-0.42985000	-4.56325600
H	9.14643700	-0.78482900	-2.83406100
H	-7.70395700	-0.57780600	-0.29401900
H	-6.24515400	2.71114800	-2.58123800
H	-7.44160000	-1.66658400	1.33020200
H	-7.00775900	-3.31550600	3.07952500
H	-4.66123100	-3.77336200	3.81326400
H	-2.79270100	-2.61098500	2.71492500
H	-3.26899800	-2.76113000	-0.73430900
H	-4.51235900	-4.77913200	-1.32053500
H	-3.72661800	-7.01292500	-0.50868300
H	-1.62734500	-7.17241700	0.74919600
H	-0.16633000	-7.17278200	2.18514400
H	2.11241100	-7.32465100	3.08111000
H	3.64483700	-5.35714500	2.93576200
H	2.85325300	-3.25805700	1.93266000
H	5.20295700	-3.41946000	-3.36258700
H	3.68104300	-5.28180600	-3.82396200
H	1.45856900	-5.36925100	-2.66119700
H	0.90048100	-3.71603000	-0.98759600
H	-9.18727700	2.49651300	-0.26703000
H	-10.53692100	1.75888000	-1.15561400
H	-9.52025600	0.76644900	-0.10018600
H	-9.05134700	-0.82800000	-2.09933800
H	-10.07900800	0.19019600	-3.11907500
H	-8.40937400	-0.16620700	-3.60992500
H	-8.05556000	2.36083200	-3.87356200
H	-9.71223500	2.62675900	-3.31529800
H	-8.37329600	3.45065300	-2.50601000

138

TS2a_-3233.805569 Hartree_Imaginary frequency: -12.12 cm-1

C	-6.76948800	0.06610800	2.64910100
C	-6.17567100	1.25750100	2.24256700
C	-5.12050800	1.30060000	1.31706700
C	-6.30335400	-1.11007000	2.05963600

C	-5.27985100	-1.12407200	1.10603500
C	-4.62761000	0.09142200	0.75567600
C	-3.44098700	0.08218000	-0.08246300
C	-2.71388700	1.30673700	-0.25505900
C	-3.19852700	2.54878700	0.36636300
C	-4.45475400	2.56681100	1.02219900
C	-4.97868700	3.80163100	1.46671400
C	-4.24852100	4.97302100	1.38444100
C	-2.93749600	4.92658200	0.89363200
C	-2.43050100	3.73768500	0.40303500
C	-0.83405700	-0.01976400	-0.98355600
C	-1.47972800	1.24320900	-0.93647700
C	-0.72433000	2.32781900	-1.57501800
C	-1.38299200	3.30218800	-2.43505200
C	-2.75666200	3.20284700	-2.76037800
C	-3.39407300	4.17076100	-3.51118200
C	-2.67133000	5.28216700	-3.97217200
C	-1.31693000	5.37576700	-3.71215300
C	-0.63234400	4.38899500	-2.96490800
C	0.81716800	4.40985500	-2.79827200
C	1.61865200	5.43285700	-3.35611800
C	2.99912500	5.37209400	-3.32958000
C	3.63360000	4.25454500	-2.76594500
C	2.87625100	3.25666200	-2.18485300
C	1.46348700	3.32797600	-2.13329400
C	0.65983900	2.32721700	-1.44019100
C	1.26345200	1.22676400	-0.68359500
C	2.42866900	1.27596600	0.12250800
C	2.85697000	2.46760500	0.85283900
C	4.12214500	2.48949300	1.49982800
C	4.49483900	3.65580600	2.20402300
C	3.65480900	4.75171700	2.29970900
C	2.38294400	4.70440100	1.71320100
C	1.99609500	3.57769900	1.01265100
C	-7.42570400	-0.85722300	4.89436400
C	-7.89159700	0.00559200	3.69840500
C	-9.15186700	-0.62977200	3.06570600
C	-8.27380300	1.39890900	4.23091900
C	7.03805800	0.13981200	2.12766800
C	6.55657700	-1.01220900	1.49798700
C	5.32971200	-1.04675700	0.82817500
C	6.24264400	1.28360000	2.07704700
C	4.97994600	1.30031700	1.46251900
C	4.50398100	0.11469100	0.84046700
C	3.18601000	0.07486800	0.24886700
C	2.66677500	-1.15961300	-0.22308100
C	3.61293700	-2.22697400	-0.53531100
C	4.91509600	-2.20197100	0.02879200
C	5.80573300	-3.25234200	-0.28306500
C	5.45222100	-4.26973500	-1.15270200
C	4.20031800	-4.24028700	-1.78403100
C	3.30770000	-3.22835300	-1.48596300
C	0.59225300	-0.02254200	-0.77904900
C	1.25501100	-1.23466600	-0.44692800
C	0.47945800	-2.45282300	-0.52038300
C	0.87483400	-3.74393600	0.04961200
C	1.64470900	-3.81451000	1.22892000
C	1.92534200	-5.03343200	1.82403600

C	1.44102000	-6.22246500	1.25796600
C	0.67124700	-6.17391700	0.10588700
C	0.37536100	-4.94675100	-0.51509900
C	-0.36591600	-4.83246700	-1.76341700
C	-0.45443200	-5.87525700	-2.70560900
C	-1.03144500	-5.67363000	-3.95058800
C	-1.50298500	-4.39788900	-4.30077200
C	-1.45203900	-3.36597800	-3.37865800
C	-0.94475400	-3.57352400	-2.07396600
C	-0.75977300	-2.43270900	-1.18200300
C	-1.58675300	-1.22159700	-1.04892900
C	-2.98302900	-1.14746800	-0.66885900
C	-3.91320400	-2.28953100	-0.61652600
C	-4.93923800	-2.33608500	0.36871600
C	-5.69623800	-3.51871300	0.51282700
C	-5.53652500	-4.60217200	-0.33409100
C	-4.64925100	-4.49266300	-1.40782200
C	-3.87499100	-3.35237400	-1.53712000
C	9.50179600	-0.25748100	1.80111000
C	8.40587700	0.10583700	2.83049300
C	8.37855800	-0.95992800	3.95060800
C	8.77667000	1.45889700	3.46554900
H	-6.49903300	2.18120600	2.69825200
H	-6.76298500	-2.04530400	2.35113100
H	-5.97888400	3.84200300	1.88223000
H	-4.67914100	5.90940200	1.72841600
H	-2.31686500	5.81815000	0.88717000
H	-1.41461200	3.73018000	0.04045200
H	-3.32248700	2.34854000	-2.41206600
H	-4.45061900	4.06775400	-3.74196600
H	-3.16599400	6.05625800	-4.55253700
H	-0.77304000	6.22168500	-4.11593500
H	1.14991400	6.28455500	-3.83509100
H	3.58582200	6.17434500	-3.76878500
H	4.71648900	4.16973500	-2.78235600
H	3.37892900	2.39745000	-1.76261800
H	5.45500500	3.70301900	2.70421100
H	3.97459400	5.63218100	2.85035100
H	1.69619900	5.53983100	1.81627700
H	1.00032900	3.53782800	0.59127300
H	-6.53284900	-0.42608400	5.36099600
H	-8.21534400	-0.91514300	5.65348500
H	-7.18164900	-1.87978200	4.58810600
H	-8.96020500	-1.64624300	2.70657400
H	-9.96213400	-0.68374500	3.80304400
H	-9.50203200	-0.03474600	2.21465700
H	-8.63850600	2.05309600	3.43063300
H	-9.07568300	1.30403100	4.97153100
H	-7.42738800	1.89297900	4.72138700
H	7.16403200	-1.90855300	1.52514400
H	6.60646700	2.18799900	2.54175600
H	6.80826600	-3.24983600	0.12971800
H	6.16161500	-5.06187200	-1.37623700
H	3.93303000	-4.99752100	-2.51545000
H	2.35977200	-3.19056200	-2.00831100
H	2.00399800	-2.89746400	1.68215400
H	2.51145300	-5.06316200	2.73828800
H	1.65353700	-7.17811400	1.72926300

H	0.27036300	-7.09308400	-0.31101700
H	-0.01480900	-6.83998400	-2.47125500
H	-1.07240600	-6.48768700	-4.66881400
H	-1.89098600	-4.21214300	-5.29841900
H	-1.78929700	-2.36991500	-3.65352800
H	-6.45844500	-3.57922800	1.28013100
H	-6.13743200	-5.49666200	-0.19446500
H	-4.56537000	-5.28171300	-2.14995700
H	-3.25701100	-3.26243700	-2.40567000
H	9.53720600	0.48187600	0.99301200
H	10.48585600	-0.28404600	2.28492200
H	9.32686900	-1.23899300	1.34845800
H	8.16575600	-1.95945100	3.55741100
H	9.34869000	-1.00186900	4.46076500
H	7.61015000	-0.72252100	4.69495100
H	8.04861900	1.76394500	4.22589800
H	9.75370800	1.38067600	3.95521000
H	8.84618200	2.25504500	2.71544400

138
TS2b_-3233.744994 Hartree_Imaginary frequency: -35.16 cm-1

C	7.18080000	-0.00724600	-1.33838200
C	6.64346200	-1.21782100	-0.89243300
C	5.50768800	-1.26561000	-0.06811700
C	6.61142300	1.18152600	-0.85002900
C	5.50366900	1.17371000	-0.00369000
C	4.87114100	-0.05918600	0.29536600
C	3.50701700	-0.06214100	0.79848100
C	2.81362000	-1.28908500	0.77902100
C	3.62402300	-2.49785500	0.95649100
C	4.96359000	-2.50134500	0.49401400
C	5.73163200	-3.67149300	0.63376200
C	5.20151100	-4.80582000	1.23127500
C	3.89131500	-4.78592400	1.73064500
C	3.11842200	-3.64343300	1.59845000
C	0.74565300	0.01360300	0.66805400
C	1.41582500	-1.24671700	0.56215100
C	0.67206500	-2.39455700	0.04739600
C	1.32860700	-3.42104600	-0.75810300
C	2.48708100	-3.15762200	-1.52388100
C	3.15797900	-4.16760800	-2.18558300
C	2.67937900	-5.48528200	-2.11201300
C	1.48490800	-5.74548300	-1.46449500
C	0.76047000	-4.71775500	-0.81964400
C	-0.62492100	-4.88804900	-0.39877800
C	-1.26557700	-6.14471300	-0.44933800
C	-2.64432600	-6.25512600	-0.41581000
C	-3.41924700	-5.08797300	-0.40173200
C	-2.80571700	-3.85352900	-0.27616900
C	-1.40491100	-3.71892000	-0.14590600
C	-0.71762100	-2.46504700	0.21421600
C	-1.39483800	-1.22779800	0.55217800
C	-2.79090200	-1.05009400	0.80305400
C	-3.59172500	-1.83206600	1.72472700
C	-5.00853700	-1.69499300	1.72308300
C	-5.74679900	-2.42832400	2.67576200
C	-5.12706300	-3.27379600	3.58218200
C	-3.73003700	-3.40511500	3.57834600
C	-2.97868400	-2.68645500	2.66713300

C	8.87262100	-1.32507600	-2.73373900
C	8.37811900	0.06733200	-2.30044600
C	9.55177000	0.80102300	-1.61017100
C	7.96017100	0.84629900	-3.56961700
C	-7.65583100	0.12674800	-0.31399600
C	-6.83021200	0.91818300	-1.12298400
C	-5.43524000	0.86911600	-1.04786500
C	-7.04512000	-0.73884600	0.59458900
C	-5.64984500	-0.81407800	0.73994300
C	-4.83769200	0.01242000	-0.08090900
C	-3.40515500	-0.00807900	0.06768100
C	-2.62052800	0.99361600	-0.55364000
C	-3.16674900	1.65827800	-1.72177800
C	-4.56529700	1.60735000	-1.97043200
C	-5.06062200	2.24516300	-3.12652700
C	-4.21671100	2.89598100	-4.01296600
C	-2.83213900	2.90674400	-3.78444600
C	-2.31995800	2.28865000	-2.65952700
C	-0.66517300	0.00039900	0.43203300
C	-1.32115200	1.19239300	0.02094000
C	-0.66139400	2.45447100	0.26885600
C	-1.31638100	3.73226900	-0.07898400
C	-2.70871000	3.92544300	-0.14750200
C	-3.25353100	5.05301000	-0.74453200
C	-2.40741100	6.02304300	-1.29658500
C	-1.03913900	5.93651600	-1.08148900
C	-0.48705600	4.84459000	-0.38419000
C	0.75380700	4.93527900	0.38856900
C	1.36860100	6.17972900	0.62769400
C	2.06634000	6.41683000	1.80462500
C	2.08156900	5.43140100	2.79522900
C	1.58346800	4.16687700	2.50808800
C	1.05637200	3.84354300	1.25317800
C	0.60853400	2.48802500	0.87569900
C	1.42742700	1.25670600	0.97924900
C	2.85895200	1.16336600	1.11676000
C	3.86969200	2.15397600	1.57726600
C	5.08147300	2.29455400	0.84404000
C	6.01427200	3.27072200	1.23902300
C	5.86619900	3.95816700	2.43750900
C	4.82642200	3.59883700	3.29820700
C	3.85320400	2.70518900	2.86643900
C	-9.58831600	-0.12049900	-1.91094000
C	-9.18371500	0.23585800	-0.46137100
C	-9.62319000	1.68513000	-0.14683600
C	-9.93749700	-0.71036600	0.49141900
H	7.08576700	-2.15395400	-1.20858800
H	7.05230600	2.13501400	-1.12280900
H	6.76554300	-3.67513100	0.30237600
H	5.81194300	-5.69860900	1.33604000
H	3.48168400	-5.66122900	2.22667000
H	2.10386100	-3.62698000	1.98227500
H	2.85949800	-2.14192700	-1.58729800
H	4.05273100	-3.93763300	-2.75679000
H	3.21322700	-6.29078300	-2.60890500
H	1.07593300	-6.74860500	-1.50938700
H	-0.66822800	-7.04426200	-0.55190600
H	-3.11583900	-7.23320500	-0.45797800

H	-4.50147100	-5.14122500	-0.47850200
H	-3.42710200	-2.97407000	-0.29107800
H	-6.82547400	-2.32508400	2.71904800
H	-5.72580600	-3.82309000	4.30359100
H	-3.23816100	-4.05625000	4.29545900
H	-1.89691500	-2.76620200	2.67107900
H	8.08852200	-1.89241500	-3.24799800
H	9.71410600	-1.21835400	-3.42737900
H	9.22178600	-1.91610100	-1.87945800
H	9.28155300	1.82193600	-1.32111300
H	9.86528100	0.26829200	-0.70529300
H	10.41251700	0.86347700	-2.28710600
H	7.63545400	1.86482300	-3.33316900
H	8.80275800	0.91686500	-4.26839400
H	7.13244100	0.34182900	-4.08079700
H	-7.29312400	1.59197400	-1.83420900
H	-7.66650100	-1.37531800	1.20853900
H	-6.12089000	2.20417700	-3.35241600
H	-4.62847500	3.37410500	-4.89758800
H	-2.16300300	3.39046600	-4.49021800
H	-1.25059100	2.28812000	-2.47879100
H	-3.37043100	3.17404200	0.26163200
H	-4.33222200	5.16063600	-0.80670500
H	-2.82127300	6.87728800	-1.82550800
H	-0.39978600	6.75710400	-1.39129200
H	1.19307900	7.00568200	-0.05357700
H	2.50439400	7.39377900	1.98984800
H	2.49316000	5.64119600	3.77776200
H	1.54425500	3.41473700	3.28116400
H	6.92677800	3.39651100	0.66458900
H	6.61294100	4.68516800	2.74473600
H	4.77671500	4.00656200	4.30388500
H	3.09200600	2.37374500	3.55929500
H	-9.28605700	-1.14416200	-2.15909300
H	-10.67573500	-0.04493400	-2.03245600
H	-9.12443400	0.55103400	-2.64076700
H	-9.15877700	2.40837200	-0.82529200
H	-10.71089500	1.78408400	-0.24754100
H	-9.34752300	1.96222600	0.87695600
H	-9.71600800	-0.49052600	1.54203000
H	-11.01764400	-0.59275000	0.34973800
H	-9.69160800	-1.76101000	0.29919300

138

TS3b_-3233.779285 Hartree_Imaginary frequency: -25.59 cm-1

C	7.72627000	0.26389300	-0.05956800
C	6.97315400	1.33378200	-0.54537000
C	5.59908500	1.22363700	-0.80267300
C	7.07294500	-0.96165100	0.11271200
C	5.70767500	-1.13125700	-0.14271600
C	4.93607600	-0.00288700	-0.53271100
C	3.48976400	-0.07271500	-0.51736700
C	2.75028100	1.13293300	-0.68444400
C	3.41110000	2.26720900	-1.33114900
C	4.82599200	2.32611900	-1.37421600
C	5.44116900	3.42470400	-2.00882300
C	4.69096000	4.43427600	-2.59027000
C	3.29121300	4.35580200	-2.58034200
C	2.66748200	3.28171200	-1.97018700

C	0.70318600	-0.07830800	-0.20927600
C	1.39056300	1.16659700	-0.28178300
C	0.65814500	2.32470600	0.22952600
C	1.32635900	3.45584300	0.88121000
C	2.69564700	3.44937300	1.23581700
C	3.33803500	4.58700500	1.68593600
C	2.62242500	5.78720600	1.81109800
C	1.25608600	5.78810100	1.59929100
C	0.56854300	4.61939900	1.19938500
C	-0.88291100	4.50752500	1.29193900
C	-1.68390300	5.53425300	1.84285700
C	-3.00412300	5.31297300	2.18935700
C	-3.55321900	4.02892800	2.04658700
C	-2.80448100	3.02569600	1.46348400
C	-1.49125200	3.25886000	0.99170100
C	-0.73355000	2.25749600	0.24416800
C	-1.40360500	1.11475500	-0.38691800
C	-2.73565800	1.15895400	-0.84657000
C	-3.43031300	2.38058100	-1.25341300
C	-4.83537900	2.45537300	-1.10678400
C	-5.50188200	3.63120200	-1.49693700
C	-4.80172900	4.70711800	-2.02336200
C	-3.41254800	4.62175900	-2.19400000
C	-2.74024900	3.46951800	-1.81940200
C	9.77212100	1.79799300	0.02100300
C	9.22246100	0.38217500	0.27601300
C	10.02533600	-0.60960600	-0.59801500
C	9.43905200	0.04384500	1.76943700
C	-7.49143100	0.23218600	0.46281200
C	-6.88272900	-1.01090500	0.27467400
C	-5.61553400	-1.13601800	-0.31293000
C	-6.81214900	1.36508200	-0.00693800
C	-5.53504500	1.28944700	-0.56902800
C	-4.88694300	0.02834200	-0.64954000
C	-3.45486400	-0.05841400	-0.88746500
C	-2.79730000	-1.32209700	-0.95220200
C	-3.72207700	-2.42781000	-1.27190000
C	-5.05734700	-2.40585200	-0.77531100
C	-5.88503200	-3.52371200	-0.99339200
C	-5.47596200	-4.58104500	-1.79461400
C	-4.25414300	-4.49144700	-2.46605300
C	-3.41222300	-3.41826500	-2.21092400
C	-0.72334000	-0.13102100	-0.38921800
C	-1.37970500	-1.39300700	-0.68806700
C	-0.59676000	-2.58602300	-0.29754600
C	-0.85363000	-4.04077400	-0.43635200
C	-0.95259500	-4.65355100	-1.70112000
C	-1.09338100	-6.02616600	-1.84325500
C	-1.11305900	-6.84556600	-0.70814400
C	-0.83521600	-6.28592300	0.52989300
C	-0.62207000	-4.90074100	0.67406200
C	0.08944900	-4.35787800	1.82685300
C	0.16615200	-4.98740000	3.07935400
C	1.03496200	-4.52151700	4.05765300
C	1.85109300	-3.41160100	3.79649000
C	1.77060600	-2.75914600	2.57542500
C	0.87875900	-3.20340600	1.57743800
C	0.60489700	-2.38928100	0.39576100

C	1.43166600	-1.26681200	-0.00064000
C	2.83946700	-1.29571200	-0.18500800
C	3.64085200	-2.52706700	-0.20310300
C	5.05602900	-2.44193700	-0.09597400
C	5.81150600	-3.63317100	-0.06543000
C	5.21435800	-4.87668100	-0.18506400
C	3.83199800	-4.95796700	-0.39455400
C	3.06983500	-3.80316100	-0.41370400
C	-8.70514900	1.28462200	2.39527000
C	-8.86245600	0.39553600	1.13926300
C	-9.46700600	-0.95113100	1.57742800
C	-9.84700300	1.06746700	0.15381500
H	7.44985400	2.29194400	-0.70067600
H	7.64441900	-1.80702800	0.47684500
H	6.52293200	3.46777900	-2.07941600
H	5.18972800	5.26729500	-3.07811300
H	2.69519400	5.12459800	-3.06398300
H	1.58590500	3.21271700	-1.98443100
H	3.26059500	2.53154500	1.15864800
H	4.39490800	4.54592400	1.93331800
H	3.12525800	6.69892500	2.12205000
H	0.70204000	6.70215300	1.78121900
H	-1.25095000	6.50721300	2.04537000
H	-3.59468600	6.11826100	2.61774600
H	-4.56315900	3.81894900	2.38707300
H	-3.23580300	2.03720400	1.36676000
H	-6.58306800	3.68668900	-1.41607500
H	-5.33473700	5.60461300	-2.32511700
H	-2.86318400	5.45123500	-2.63025800
H	-1.66685900	3.39856300	-1.95932800
H	9.26130600	2.54851500	0.63494000
H	10.83727300	1.83159700	0.27585800
H	9.67426200	2.08858000	-1.03119700
H	9.88465000	-0.39211800	-1.66283200
H	11.09607900	-0.53663400	-0.37146600
H	9.71861700	-1.64633200	-0.42518400
H	9.10814400	-0.97196500	2.00852200
H	10.50264100	0.11872900	2.02722800
H	8.88195600	0.73674000	2.41017100
H	-7.39521900	-1.91034300	0.59002900
H	-7.27204400	2.34139900	0.10039000
H	-6.89723700	-3.52279200	-0.60300800
H	-6.13855800	-5.42583100	-1.96148900
H	-3.96196400	-5.24650100	-3.18982300
H	-2.51364800	-3.29538100	-2.78962100
H	-0.84281000	-4.04030600	-2.58649800
H	-1.16190700	-6.46010100	-2.83704300
H	-1.24783300	-7.91931100	-0.80432200
H	-0.68035700	-6.93596000	1.38547300
H	-0.46385100	-5.84795100	3.28473200
H	1.08105700	-5.01810600	5.02297900
H	2.53519400	-3.04637900	4.55736600
H	2.37459400	-1.87707600	2.39412800
H	6.89244900	-3.58118000	0.00250800
H	5.82333700	-5.77633900	-0.16437100
H	3.35200900	-5.91845300	-0.55933700
H	2.01405100	-3.88973500	-0.63054600
H	-8.31992300	2.27829500	2.14386200

H	-9.67370400	1.41718200	2.89293300
H	-8.01207000	0.82791700	3.11085100
H	-8.82387300	-1.46975000	2.29743500
H	-10.43541400	-0.78073500	2.06083400
H	-9.63575500	-1.61732200	0.72364300
H	-9.96726100	0.46051500	-0.75060300
H	-10.83223000	1.18579900	0.62108000
H	-9.50315200	2.06064800	-0.15289200

138

TS3a_-3233.784363 Hartree_Imaginary frequency: -23.81 cm-1

C	-6.94990600	-1.17684200	1.56377500
C	-5.96003900	-2.16020700	1.58577900
C	-4.61181700	-1.86681400	1.33210100
C	-6.54410100	0.14610200	1.34390400
C	-5.21230400	0.49172700	1.09732700
C	-4.24006800	-0.53923600	0.99747000
C	-2.93520800	-0.25932600	0.43440600
C	-2.05803500	-1.34578800	0.17533500
C	-2.26593600	-2.57360600	0.94858600
C	-3.55122000	-2.86331500	1.46940800
C	-3.74216800	-4.06707100	2.17910100
C	-2.69433800	-4.94547400	2.40075400
C	-1.40625300	-4.62028300	1.95135800
C	-1.19911600	-3.44718000	1.24784900
C	-0.59129400	0.17163600	-1.00845600
C	-0.97689600	-1.15771600	-0.73167600
C	-0.22237500	-2.19905600	-1.42815100
C	-0.87378600	-3.48072200	-1.71516800
C	-2.27829400	-3.63480800	-1.72780100
C	-2.86694600	-4.88391200	-1.79191900
C	-2.06041200	-6.03110800	-1.85049000
C	-0.68808600	-5.89730600	-1.96383100
C	-0.07417400	-4.62513500	-1.96286400
C	1.28899100	-4.40550300	-2.42639500
C	2.07633400	-5.45197100	-2.95229800
C	3.16840700	-5.19689000	-3.76570800
C	3.45208200	-3.87605700	-4.13444800
C	2.73285500	-2.84232200	-3.56032500
C	1.73402700	-3.06078400	-2.59965800
C	1.08830400	-1.96692200	-1.87152300
C	1.73124700	-0.67869000	-1.48577200
C	3.08617000	-0.41528000	-1.06520900
C	4.36279600	-1.00174400	-1.50352000
C	5.35205200	-1.31384200	-0.53464100
C	6.55996200	-1.90381200	-0.95722800
C	6.85832700	-2.04143600	-2.30508700
C	5.98264300	-1.50792500	-3.25855300
C	4.76209000	-0.98933200	-2.85318300
C	-8.69920400	-2.98680600	2.02159100
C	-8.43882500	-1.48658000	1.79157900
C	-8.93987900	-0.71563800	3.03523900
C	-9.24806900	-1.04219500	0.55081500
C	6.04294800	-0.38775500	3.07189600
C	5.24813800	0.76145400	3.13831100
C	4.39389000	1.15778900	2.09887300
C	6.02105100	-1.12104900	1.88228700
C	5.20145400	-0.75783500	0.80972200
C	4.30918400	0.34070700	0.94363500

C	3.25273500	0.54804800	-0.02332800
C	2.35352000	1.64606200	0.11537100
C	2.53332700	2.58874200	1.22842100
C	3.55944500	2.35902600	2.18804300
C	3.73401200	3.29121900	3.23319400
C	2.90060200	4.38585400	3.38338700
C	1.83629300	4.56492800	2.49183200
C	1.66036000	3.67958500	1.44308000
C	0.82159100	0.38463400	-1.22813300
C	1.25841400	1.66644700	-0.77409500
C	0.40744400	2.77602600	-1.21930200
C	0.97612900	4.02026600	-1.71843300
C	2.36800200	4.27451500	-1.68154400
C	2.89007200	5.49355000	-2.06806700
C	2.03149900	6.51044100	-2.51492400
C	0.67339900	6.27002300	-2.60809500
C	0.11109000	5.02491000	-2.24286800
C	-1.30007800	4.72093400	-2.45196400
C	-2.17372700	5.61546400	-3.11354400
C	-3.48381600	5.27594400	-3.39501900
C	-3.96791100	4.00492400	-3.04438200
C	-3.14362600	3.11743200	-2.38271900
C	-1.81341700	3.45863300	-2.04046800
C	-0.95836000	2.54181200	-1.31012700
C	-1.44233200	1.27634900	-0.73356700
C	-2.60454900	1.07423400	0.04122500
C	-3.43040300	2.15894800	0.59604800
C	-4.75285600	1.88120400	1.03078300
C	-5.54928500	2.94084200	1.51143100
C	-5.05464300	4.23119300	1.61234100
C	-3.71947300	4.48644200	1.27699800
C	-2.92556600	3.46256300	0.78846000
C	7.91575700	0.35465400	4.58159700
C	6.91316000	-0.78269200	4.27709200
C	6.00726300	-1.01482600	5.50890000
C	7.71484100	-2.07283000	4.02343400
H	-6.23437300	-3.18836300	1.78031300
H	-7.29367700	0.92908100	1.35116600
H	-4.71580800	-4.29251900	2.60141100
H	-2.86523800	-5.86372400	2.95607400
H	-0.56822800	-5.27809800	2.16313100
H	-0.19850200	-3.19080500	0.92094200
H	-2.90848300	-2.75603100	-1.67069900
H	-3.94946600	-4.97195300	-1.78328600
H	-2.51195900	-7.01940000	-1.85911300
H	-0.08485600	-6.78825200	-2.09961900
H	1.78752500	-6.48210900	-2.77471100
H	3.75011300	-6.02078300	-4.17006100
H	4.23058900	-3.65687800	-4.85909300
H	2.89860100	-1.83053600	-3.88214400
H	7.31235300	-2.16768800	-0.22102100
H	7.80615300	-2.47619900	-2.61026900
H	6.26231400	-1.47792800	-4.30800700
H	4.12346200	-0.49945100	-3.57833200
H	-8.38544500	-3.58987600	1.16200300
H	-9.77119500	-3.15644300	2.17186900
H	-8.17817200	-3.35768900	2.91159300
H	-8.37860400	-1.01178400	3.92862100

H	-10.00185700	-0.92593500	3.21204500
H	-8.83027400	0.36712000	2.91505700
H	-9.14232500	0.03050700	0.35866100
H	-10.31482500	-1.25229100	0.69610000
H	-8.91244200	-1.57723800	-0.34476600
H	5.26695400	1.34545000	4.04997900
H	6.65315400	-1.99324000	1.77426600
H	4.55057200	3.16155000	3.93427300
H	3.06648100	5.08863900	4.19525400
H	1.14708200	5.39568200	2.61466200
H	0.82225300	3.83179000	0.78010200
H	3.03928000	3.49859200	-1.33747300
H	3.96228900	5.66173200	-2.02017400
H	2.43124400	7.47904900	-2.80247200
H	0.03236200	7.06289700	-2.97596300
H	-1.81178000	6.58468800	-3.43676600
H	-4.12766900	5.98456300	-3.90886000
H	-4.98502900	3.71655400	-3.29423000
H	-3.51865100	2.13540300	-2.12290100
H	-6.55577600	2.73879500	1.86150900
H	-5.68854400	5.02717500	1.99354100
H	-3.29781700	5.47867800	1.41028700
H	-1.88690800	3.66602700	0.56589100
H	8.57064800	0.53680600	3.72193500
H	8.54388300	0.08941200	5.44080100
H	7.40681600	1.29464200	4.81865700
H	5.43973700	-0.11653000	5.77288100
H	6.61234400	-1.29557000	6.37967200
H	5.28885300	-1.81973100	5.31718700
H	7.05688700	-2.92532900	3.81998600
H	8.31041200	-2.31754100	4.90992100
H	8.40684300	-1.96277700	3.18064300

138

TS4b_-3233.742475 Hartree_Imaginary frequency: -12.68 cm-1

C	-7.47306200	-0.46008700	-0.82544100
C	-6.61102500	-1.50099600	-1.17858500
C	-5.21762200	-1.34092800	-1.18255100
C	-6.90430600	0.79080500	-0.54710100
C	-5.52258500	1.00241700	-0.55465500
C	-4.66160800	-0.10378500	-0.77609500
C	-3.25408700	-0.00246000	-0.45015500
C	-2.46502900	-1.17794400	-0.46540200
C	-2.92126000	-2.26272100	-1.33831300
C	-4.29771500	-2.37226200	-1.65869600
C	-4.72363000	-3.42617600	-2.49093800
C	-3.81999500	-4.33593300	-3.01642600
C	-2.45079700	-4.18940000	-2.75069600
C	-2.01246700	-3.16076300	-1.93498900
C	-0.64191200	0.03954600	0.60471400
C	-1.25304300	-1.19883200	0.28773600
C	-0.62953300	-2.39601600	0.81140800
C	-1.44730800	-3.61319600	0.91122500
C	-2.83545800	-3.58490800	1.15171500
C	-3.60415300	-4.73078600	1.03998400
C	-2.99670000	-5.94312800	0.68141600
C	-1.61915300	-6.01242400	0.55521000
C	-0.81857400	-4.86313900	0.72373900
C	0.61356400	-4.92191800	0.98221200

C	1.31683200	-6.14268300	0.97760500
C	2.48349200	-6.30626100	1.70930500
C	2.89555200	-5.26647800	2.54843600
C	2.25037500	-4.04070900	2.47866900
C	1.22026600	-3.76668300	1.57121800
C	0.70360100	-2.41448900	1.25165500
C	1.50638000	-1.15502000	1.15087300
C	2.93825400	-1.03847300	1.09272500
C	4.04021100	-1.88076200	1.62222100
C	5.16544700	-2.14865700	0.79887900
C	6.19654200	-2.96654600	1.29741900
C	6.22240000	-3.34908800	2.63290200
C	5.25627500	-2.83972400	3.50949200
C	4.18902600	-2.10921100	3.00258500
C	-9.44797800	-2.06415500	-1.09633200
C	-9.00022300	-0.63030400	-0.75824400
C	-9.67095400	0.33310600	-1.76525700
C	-9.48618800	-0.29960500	0.67226600
C	6.79189000	-0.36895500	-2.14311300
C	6.28064900	0.90964600	-1.86857100
C	5.30413400	1.12368300	-0.89042600
C	6.37405300	-1.43172300	-1.32945000
C	5.40384100	-1.25191000	-0.33858500
C	4.77065800	0.00562800	-0.21017900
C	3.51058200	0.11757300	0.50033800
C	2.84342700	1.35795000	0.51527600
C	3.74560400	2.51935300	0.44500000
C	4.90031300	2.43693400	-0.37705100
C	5.71984300	3.57271200	-0.51381400
C	5.46332500	4.73204600	0.20631800
C	4.40333200	4.76218400	1.12108700
C	3.56025800	3.66733300	1.23609700
C	0.77669300	0.06826500	0.85958100
C	1.43188100	1.34613200	0.65122600
C	0.60596100	2.54600300	0.59551900
C	0.95848500	3.87095200	0.07038000
C	1.78237900	4.01210000	-1.06700000
C	2.05360300	5.25701100	-1.60689300
C	1.50311100	6.40882300	-1.02541100
C	0.63061800	6.28713800	0.04481400
C	0.31326100	5.02668800	0.59037400
C	-0.72922600	4.84981700	1.59690100
C	-1.22584100	5.88699600	2.40623900
C	-2.30913500	5.67817200	3.24845300
C	-2.92533900	4.41832000	3.30359200
C	-2.44383000	3.37779300	2.52623000
C	-1.33890000	3.56993400	1.67313900
C	-0.73462000	2.45205500	0.96684100
C	-1.41063300	1.24142000	0.55634100
C	-2.71475000	1.23574200	-0.01112000
C	-3.49653700	2.43458800	-0.36089200
C	-4.90813600	2.33082400	-0.48742200
C	-5.65968100	3.49781600	-0.73387500
C	-5.04554300	4.72318100	-0.93992100
C	-3.64734300	4.79423800	-0.97348500
C	-2.89415600	3.66459700	-0.70092700
C	9.06408900	0.32281400	-2.98807900
C	7.81719900	-0.54577000	-3.27574500

C	7.17649800	-0.09961600	-4.61111800
C	8.27677000	-2.00744400	-3.42987400
H	-7.01726500	-2.47093500	-1.43414000
H	-7.55883000	1.62281900	-0.31276700
H	-5.77165900	-3.50354300	-2.76211200
H	-4.16975100	-5.13746400	-3.66136500
H	-1.73050300	-4.87191800	-3.19269600
H	-0.95163900	-3.03975100	-1.74685400
H	-3.31257300	-2.64426400	1.40224900
H	-4.67468800	-4.68329500	1.21662500
H	-3.59751800	-6.83996600	0.55726500
H	-1.15431200	-6.97794900	0.38737700
H	0.90096600	-6.99280700	0.44671500
H	3.01051500	-7.25636300	1.69691500
H	3.71731100	-5.39849400	3.24553900
H	2.51283100	-3.27473600	3.17762600
H	7.04370500	-3.19923700	0.65912900
H	7.04436400	-3.95149700	3.00974600
H	5.34120200	-3.00381400	4.58003800
H	3.45320500	-1.68526500	3.68059000
H	-9.02154300	-2.79646500	-0.40137600
H	-10.53900200	-2.13654200	-1.02623200
H	-9.16295600	-2.34954300	-2.11544200
H	-9.34267500	0.11697100	-2.78821600
H	-10.76196300	0.22710400	-1.72571700
H	-9.42995400	1.37941300	-1.55092800
H	-9.23599100	0.72664300	0.96073800
H	-10.57537200	-0.41000000	0.74085400
H	-9.02715100	-0.97388600	1.40417100
H	6.64921800	1.76054500	-2.43200700
H	6.81412700	-2.41456500	-1.44803200
H	6.60582500	3.51863900	-1.13900200
H	6.11727900	5.59293900	0.09742400
H	4.23371300	5.64048000	1.73692200
H	2.73650300	3.69444200	1.93982600
H	2.20228100	3.12525000	-1.52573200
H	2.69091000	5.33549700	-2.48289600
H	1.72171200	7.38949700	-1.43892200
H	0.13956800	7.17333400	0.43482200
H	-0.74927400	6.86239200	2.38107100
H	-2.67597000	6.49124300	3.86893800
H	-3.77175600	4.25335300	3.96430200
H	-2.90419900	2.39647700	2.57667600
H	-6.73812700	3.42938900	-0.83009900
H	-5.64717400	5.60632000	-1.13698200
H	-3.14541600	5.72453100	-1.22359800
H	-1.81736100	3.72569000	-0.78428500
H	9.53190100	0.02979800	-2.04143200
H	9.80454400	0.20308300	-3.78837200
H	8.81480800	1.38693600	-2.92296500
H	6.86390500	0.94937100	-4.58128900
H	7.89243400	-0.21249900	-5.43451500
H	6.29257500	-0.70536900	-4.84059200
H	7.43537900	-2.67614300	-3.64372100
H	8.98435500	-2.08638100	-4.26268200
H	8.78564300	-2.37105900	-2.52977100

138

TS4a_-3233.780376 Hartree_Imaginary frequency: -32.85 cm-1

C	-7.46797700	0.23855400	1.14800800
C	-6.83134100	-1.00193900	1.06489600
C	-5.57926500	-1.15657400	0.45101700
C	-6.84387100	1.33371800	0.53375400
C	-5.59337500	1.23014700	-0.08123200
C	-4.90645300	-0.01091600	-0.03977100
C	-3.49366300	-0.07539700	-0.35327800
C	-2.80375700	-1.30607500	-0.22494600
C	-3.63486100	-2.51592700	-0.28756200
C	-4.97772500	-2.46133000	0.17120000
C	-5.74330000	-3.64548900	0.18044100
C	-5.23775400	-4.83474500	-0.32060000
C	-3.96543100	-4.85202900	-0.90793100
C	-3.18861400	-3.70648500	-0.89962800
C	-0.67158300	-0.08035800	-0.46814600
C	-1.37816600	-1.28776400	-0.19641500
C	-0.57370500	-2.46981200	-0.00098200
C	-0.94295000	-3.63225400	0.80725600
C	-1.70256300	-3.48267300	1.98322400
C	-1.93646700	-4.56777800	2.81416000
C	-1.41396900	-5.82923600	2.49150000
C	-0.66454500	-5.99448000	1.33417000
C	-0.42322700	-4.90962600	0.47646900
C	0.22164300	-5.00939600	-0.82643000
C	0.21103400	-6.18526900	-1.59965000
C	0.62015200	-6.17835500	-2.92565200
C	1.00924300	-4.96922700	-3.52342100
C	1.06033000	-3.80699700	-2.76947000
C	0.74471700	-3.81629600	-1.39037100
C	0.66735600	-2.54538100	-0.65406100
C	1.52143800	-1.34945100	-0.64131900
C	2.97018700	-1.29648400	-0.45647500
C	3.90735200	-2.44161900	-0.51817400
C	5.17334500	-2.39196900	0.13746000
C	5.91193200	-3.58047800	0.32259900
C	5.53034900	-4.78079900	-0.25026500
C	4.41941900	-4.78196900	-1.09679600
C	3.65274900	-3.63559200	-1.21769800
C	-9.35582000	-0.87074700	2.47235500
C	-8.82205800	0.43325600	1.85089500
C	-9.86417300	0.93964900	0.82621500
C	-8.66438500	1.47545600	2.98254200
C	7.70661400	0.25895800	1.04858800
C	6.86630200	1.36819400	1.10211600
C	5.48494700	1.28219200	0.87215000
C	7.11083900	-0.97401000	0.78012800
C	5.74240900	-1.10761900	0.51125300
C	4.90937100	0.03605000	0.48702200
C	3.52273200	-0.06306500	0.05348400
C	2.74993000	1.12770900	0.10420000
C	3.23124900	2.28811000	0.86394200
C	4.61001000	2.41696100	1.14819900
C	5.06600800	3.57590300	1.81553600
C	4.18834800	4.55170100	2.25274500
C	2.80938800	4.36278500	2.08684000
C	2.34704100	3.24549200	1.41753000
C	0.77489400	-0.11879800	-0.52977700
C	1.45367400	1.12013500	-0.46693900

C	0.74419000	2.34375900	-0.78348000
C	1.40781600	3.51511200	-1.34634500
C	2.61821400	3.39851500	-2.06416900
C	3.17867600	4.49216000	-2.70050100
C	2.53740300	5.73948400	-2.64600000
C	1.34651300	5.87395300	-1.95088500
C	0.76491400	4.77887100	-1.28151700
C	-0.44235800	4.89102100	-0.47133700
C	-0.91060600	6.13298200	0.00698900
C	-2.00996100	6.21544700	0.84393900
C	-2.65579500	5.04057700	1.25845400
C	-2.22146900	3.81263400	0.79454600
C	-1.13908100	3.70609300	-0.10871600
C	-0.62522200	2.40191600	-0.53828600
C	-1.38376200	1.15999200	-0.55302700
C	-2.80296800	1.11959500	-0.67775500
C	-3.61991700	2.21018000	-1.22576700
C	-4.98656400	2.31093700	-0.86022700
C	-5.74798700	3.38832000	-1.35498200
C	-5.19953300	4.31759700	-2.22512500
C	-3.87685400	4.16265400	-2.66224100
C	-3.10826000	3.11937500	-2.17505600
C	9.95457500	-0.13790000	-0.00489400
C	9.22520900	0.34472500	1.27065900
C	9.62189000	-0.55738800	2.46242400
C	9.69422200	1.77879500	1.57842700
H	-7.30532100	-1.87460300	1.49429200
H	-7.33819500	2.29868800	0.55003600
H	-6.77074500	-3.61630500	0.52795200
H	-5.85093400	-5.73175600	-0.31118700
H	-3.58601500	-5.75357200	-1.38030900
H	-2.22655100	-3.72053300	-1.39665900
H	-2.09495600	-2.50370700	2.23804800
H	-2.51768900	-4.43497400	3.72232700
H	-1.58887100	-6.67594300	3.14949800
H	-0.25030800	-6.96940500	1.09294400
H	-0.18260900	-7.09872500	-1.16350800
H	0.58547200	-7.09255900	-3.51128200
H	1.25669200	-4.93572300	-4.58076900
H	1.35225000	-2.86756100	-3.23191500
H	6.80863100	-3.55319800	0.93124100
H	6.11381800	-5.68183000	-0.08235800
H	4.13876700	-5.66747700	-1.66040600
H	2.85026500	-3.65977500	-1.91646200
H	-8.66891800	-1.27234800	3.22601600
H	-10.31457300	-0.67819900	2.96662600
H	-9.52526100	-1.64325300	1.71365300
H	-9.99411600	0.21599200	0.01355700
H	-10.83633200	1.08769100	1.31218400
H	-9.56606400	1.89389300	0.37957000
H	-8.32675300	2.44419400	2.59997400
H	-9.62285500	1.63274500	3.49219500
H	-7.93341100	1.13549700	3.72488900
H	7.28976600	2.33543300	1.33070000
H	7.74237800	-1.85059900	0.71598400
H	6.12015700	3.69388200	2.03749500
H	4.56663500	5.43155200	2.76606100
H	2.09893000	5.07951100	2.48860800

H	1.27825000	3.10089900	1.33819600
H	3.10185000	2.43058500	-2.13185000
H	4.10522900	4.37742400	-3.25600700
H	2.96523400	6.59662000	-3.15879600
H	0.84525300	6.83645500	-1.93988300
H	-0.37317200	7.03966500	-0.25070400
H	-2.34621400	7.18317800	1.20591300
H	-3.48800600	5.08846500	1.95504000
H	-2.70865200	2.91127200	1.14456000
H	-6.79832000	3.46590000	-1.09346100
H	-5.80885500	5.13531400	-2.60020100
H	-3.45442300	4.85034900	-3.38927800
H	-2.09236600	2.99503600	-2.53154900
H	9.69096400	0.48895900	-0.86426900
H	11.04149100	-0.08839900	0.13494100
H	9.69659100	-1.17233800	-0.25425700
H	9.35700900	-1.60511200	2.28668800
H	10.70464200	-0.51117000	2.63126100
H	9.11782300	-0.23397900	3.38014500
H	9.22929200	2.17128600	2.49011200
H	10.77932500	1.78730400	1.73004800
H	9.47003600	2.46544700	0.75412800

42
[6]helicene_-1000.488513 Hartree

C	-1.1658220	3.5853280	-0.4047650
C	-2.2955560	2.9142680	-0.7746250
C	-2.3975350	1.5087190	-0.5626660
C	-3.6341630	0.8392880	-0.8323170
C	-3.8117510	-0.4716470	-0.5108800
C	-2.8065360	-1.1774600	0.2217430
C	-3.0600850	-2.4800580	0.7163680
C	-2.1536380	-3.1193650	1.5357540
C	-0.9699520	-2.4525800	1.9092830
C	-0.6863150	-1.1960740	1.4126750
C	-1.5632860	-0.5352830	0.5164460
C	-1.2929030	0.7841450	-0.0393000
C	0.0000010	1.4451370	0.0000000
C	1.2929040	0.7841440	0.0393000
C	1.5632860	-0.5352840	-0.5164460
C	2.8065350	-1.1774630	-0.2217430
C	3.0600820	-2.4800610	-0.7163670
C	2.1536340	-3.1193670	-1.5357530
C	0.9699500	-2.4525810	-1.9092830
C	0.6863140	-1.1960750	-1.4126750
C	3.8117500	-0.4716500	0.5108790
C	3.6341650	0.8392850	0.8323150
C	2.3975370	1.5087180	0.5626650
C	2.2955590	2.9142670	0.7746250
C	0.0000010	2.8732060	0.0000010
C	1.1658250	3.5853270	0.4047660
H	-1.1216710	4.6706240	-0.4506920
H	-3.1610810	3.4488190	-1.1579160
H	-4.4321910	1.4070280	-1.3046630
H	-4.7448870	-0.9796860	-0.7416520
H	-4.0026770	-2.9576510	0.4587490
H	-2.3631420	-4.1156060	1.9155640
H	-0.2749670	-2.9253860	2.5976890
H	0.2208610	-0.6987870	1.7302790

```
H	4.0026740	-2.9576550	-0.4587470
H	2.3631380	-4.1156090	-1.9155630
H	0.2749640	-2.9253860	-2.5976890
H	-0.2208610	-0.6987860	-1.7302800
H	4.7448860	-0.9796900	0.7416510
H	4.4321940	1.4070250	1.3046600
H	3.1610840	3.4488170	1.1579150
H	1.1216740	4.6706230	0.4506950
42
TS_[6]helicene_-1000.427538 Hartree_Imaginary frequency: -40.80 cm-1
C	-0.0000020	1.3635460	-0.5236470
C	1.3157630	0.7750130	-0.2082960
C	-1.3157660	0.7750090	-0.2082960
C	1.7266320	-0.6288330	-0.1845770
C	-1.7266320	-0.6288370	-0.1845780
C	1.4093320	-1.5427050	-1.2100950
C	-1.4093240	-1.5427090	-1.2100930
C	-0.0000030	2.7803170	-0.7713730
C	1.2018450	3.5475940	-0.7591910
C	-1.2018530	3.5475920	-0.7591890
C	2.3111330	3.0354740	-0.1602330
C	-2.3111400	3.0354690	-0.1602310
C	2.3383360	1.6676580	0.2244220
C	-2.3383400	1.6676530	0.2244230
C	3.3861070	1.2119320	1.0929520
C	-3.3861110	1.2119240	1.0929510
C	3.4732020	-0.0901610	1.4758800
C	-3.4732060	-0.0901690	1.4758760
C	2.7037460	-1.0665090	0.7682670
C	-2.7037470	-1.0665160	0.7682630
C	3.0422790	-2.4376280	0.8463620
C	-3.0422770	-2.4376350	0.8463580
C	2.5803090	-3.3384450	-0.0941210
C	-2.5803000	-3.3384510	-0.0941230
C	1.8163600	-2.8645140	-1.1744300
C	-1.8163470	-2.8645200	-1.1744290
H	-0.9117300	-1.1653230	-2.0899460
H	-1.5564180	-3.5296880	-1.9931630
H	-2.8696790	-4.3844840	-0.0423770
H	-3.7405720	-2.7557640	1.6169620
H	4.2120060	-0.4184000	2.2023680
H	-4.2120100	-0.4184110	2.2023620
H	4.0725530	1.9567210	1.4882170
H	-4.0725590	1.9567120	1.4882160
H	3.1761390	3.6587150	0.0520170
H	-3.1761470	3.6587090	0.0520200
H	1.1568900	4.5935720	-1.0506240
H	-1.1569000	4.5935700	-1.0506220
H	0.9117410	-1.1653200	-2.0899510
H	1.5564390	-3.5296830	-1.9931680
H	2.8696910	-4.3844770	-0.0423750
H	3.7405720	-2.7557560	1.6169690
48
a-fused [6]helicene_-1154.139406 Hartree
C	-1.0455610	-3.0536330	2.1197140
C	0.1482700	-2.3390420	2.2933140
C	0.3026790	-1.1028230	1.6942620
C	-0.7097560	-0.5359950	0.8867830
```

C	-1.9434910	-1.2308320	0.7667970
C	-2.0750130	-2.4944620	1.3839240
C	-0.5523360	0.7909050	0.2810210
C	-1.7271060	1.5082050	-0.0346910
C	-2.9821300	0.7846870	-0.2430380
C	-3.0614760	-0.5993630	0.0687450
C	0.7291370	1.4487670	0.1282900
C	0.7402240	2.8750720	0.1824900
C	-0.4795560	3.5882670	0.0509760
C	-1.6593690	2.9248900	-0.1504840
C	2.0034360	0.7782330	-0.0701020
C	3.1886730	1.4864180	0.2620790
C	3.1382800	2.8881970	0.5219410
C	1.9659410	3.5729660	0.3898770
C	2.1562330	-0.5388080	-0.6728390
C	3.4250990	-1.1950400	-0.6060220
C	4.5519160	-0.5035620	-0.0618030
C	4.4473680	0.8058300	0.2970750
C	3.5717210	-2.4985470	-1.1398570
C	2.5259310	-3.1262110	-1.7832110
C	1.3021330	-2.4454900	-1.9396360
C	1.1264560	-1.1867790	-1.4006450
C	-4.1030950	1.4084140	-0.8352710
C	-5.2557370	0.7005860	-1.1300100
C	-5.3212270	-0.6714840	-0.8495050
C	-4.2381580	-1.3039590	-0.2633270
H	-1.1783480	-4.0249820	2.5882110
H	0.9469790	-2.7438020	2.9084510
H	1.2175830	-0.5489470	1.8603830
H	-3.0163430	-3.0286350	1.3183620
H	-0.4554370	4.6747930	0.0764740
H	-2.5653500	3.4974520	-0.3106720
H	4.0644390	3.4088620	0.7519750
H	1.9384520	4.6560340	0.4794900
H	5.5062530	-1.0214480	-0.0052830
H	5.3223790	1.3618070	0.6248770
H	4.5397580	-2.9872340	-1.0559880
H	2.6530370	-4.1238370	-2.1945720
H	0.4902810	-2.9082600	-2.4937910
H	0.1845250	-0.6768700	-1.5552090
H	-4.0576630	2.4571100	-1.1062900
H	-6.0967740	1.2057800	-1.5968610
H	-6.2129100	-1.2404290	-1.0979250
H	-4.2977750	-2.3698440	-0.0727170

48

TS_a-fused [6]helicene_-1154.080363 Hartree_Imaginary frequency: -33.84 cm-1

C	-0.5120160	1.3431390	-0.7374480
C	0.6772790	0.4840620	-0.7344700
C	-1.7970080	1.0480990	-0.0744170
C	0.7482390	-0.9867080	-0.7070530
C	-2.5180140	-0.2163140	0.0306840
C	0.0046370	-1.7726320	-1.6027110
C	-2.6862480	-1.0962910	-1.0599160
C	-0.2799000	2.7269760	-1.0469030
C	0.9960920	3.1726860	-1.4841760
C	-1.2117030	3.7459280	-0.6892120
C	2.0983890	2.4109720	-1.2250460
C	-2.1749120	3.4698100	0.2319540

C	1.9489320	1.1059390	-0.6876920
C	-2.4217210	2.1223160	0.6190550
C	3.0502750	0.4565390	0.0341850
C	-3.2952970	1.8587210	1.7260710
C	2.8727940	-0.8473470	0.5682160
C	-3.6153750	0.5870210	2.0902950
C	1.7678650	-1.6412900	0.0390480
C	-3.3173090	-0.4829680	1.1896130
C	1.7959150	-3.0500910	0.0674580
C	-3.9619430	-1.7355230	1.3223220
C	0.9355940	-3.8061660	-0.7144990
C	-3.9770560	-2.6406250	0.2789110
C	0.0769120	-3.1568700	-1.6074530
C	-3.3928340	-2.2796890	-0.9486520
C	3.8280980	-1.3618970	1.4649220
C	4.2268990	1.1610320	0.3696400
C	4.9667990	-0.6428060	1.7929440
C	5.1753800	0.6207510	1.2241790
H	-0.5797380	-1.2686730	-2.3558120
H	-2.3239090	-0.7815500	-2.0277750
H	1.1009250	4.1903480	-1.8510930
H	-1.0202470	4.7658530	-1.0119870
H	3.0880630	2.8176920	-1.3999450
H	-2.7695030	4.2613140	0.6810880
H	-3.6536270	2.7096170	2.3003530
H	-4.2124920	0.3865070	2.9761020
H	2.5588770	-3.5573440	0.6483180
H	-4.5139140	-1.9401310	2.2367310
H	0.9891790	-4.8910900	-0.6895300
H	-4.5004160	-3.5874170	0.3802330
H	-0.5184260	-3.7268790	-2.3150790
H	-3.5092240	-2.9211060	-1.8178820
H	3.6708050	-2.3380180	1.9125100
H	4.3843250	2.1659660	-0.0055170
H	5.6910290	-1.0579530	2.4883880
H	6.0657420	1.1922190	1.4718040

48

d-fused [6]helicene_-1154.137855 Hartree

C	4.2293160	-0.0212970	2.0190980
C	2.9804030	0.5975770	2.2208780
C	1.8589790	0.1485170	1.5514220
C	1.9216810	-0.9357930	0.6413760
C	3.1725090	-1.6152450	0.5136040
C	4.3139260	-1.1192260	1.1882870
C	0.7639820	-1.4334390	-0.0938920
C	0.8526260	-2.7371700	-0.6525910
C	2.1209990	-3.3956130	-0.7511760
C	3.2480710	-2.8299290	-0.2406840
C	-0.4890710	-0.7234230	-0.2227970
C	-1.6710390	-1.4782330	-0.4252030
C	-1.5545150	-2.8149150	-0.8952280
C	-0.3271600	-3.3890280	-1.0933740
C	-0.6173330	0.7333010	-0.1854400
C	-1.8316690	1.2950230	0.2563450
C	-3.0154070	0.4517080	0.3884420
C	-2.9636100	-0.8871780	-0.0930290
C	0.4190870	1.6224030	-0.6831170
C	0.3249280	3.0227530	-0.4152690

C	-0.8391510	3.5249360	0.2326570
C	-1.8960390	2.7030130	0.4972310
C	1.3527650	3.8972730	-0.8485090
C	2.4201380	3.4276640	-1.5817720
C	2.4740280	2.0611950	-1.9286740
C	1.5016170	1.1862750	-1.4914160
C	-4.2443330	0.9454930	0.8810100
C	-5.3954630	0.1785040	0.8604630
C	-5.3556050	-1.1225550	0.3379900
C	-4.1590090	-1.6406570	-0.1238620
H	5.1114910	0.3456030	2.5365820
H	2.8929230	1.4311460	2.9122080
H	0.9080960	0.6299870	1.7404930
H	5.2599350	-1.6399930	1.0586830
H	2.1608700	-4.3611320	-1.2495940
H	4.2116740	-3.3239220	-0.3387650
H	-2.4441920	-3.3814250	-1.1435670
H	-0.2481560	-4.3840550	-1.5241030
H	-0.9027430	4.5886450	0.4488920
H	-2.8050780	3.1378320	0.8950630
H	1.2673400	4.9553490	-0.6116330
H	3.1996150	4.1068640	-1.9160140
H	3.2844070	1.6923970	-2.5513770
H	1.5588460	0.1472130	-1.7882590
H	-4.3008910	1.9492360	1.2859250
H	-6.3257270	0.5867150	1.2457370
H	-6.2557020	-1.7304370	0.3104880
H	-4.1466210	-2.6616160	-0.4873740

48

TS_d-fused [6]helicene_-1154.079706 Hartree_Imaginary frequency: -34.15 cm-1

C	0.4609570	-0.7311610	-0.3629040
C	-0.7572190	-1.5039010	-0.0981890
C	0.6194480	0.7179700	-0.0431680
C	-2.1529280	-1.0913170	-0.2379380
C	-0.3516540	1.7995540	-0.1368830
C	-2.6243460	-0.3554930	-1.3421730
C	-1.1372740	2.0355000	-1.2877410
C	1.6703250	-1.4772260	-0.4784360
C	1.6923700	-2.8846580	-0.3022590
C	2.9669590	-0.8101020	-0.5035160
C	0.6120210	-3.5211190	0.2419210
C	3.0650030	0.4210440	0.1828530
C	-0.5991880	-2.8186900	0.4315120
C	1.8363130	1.0931550	0.5724760
C	-1.6396510	-3.4170330	1.2221490
C	1.8493550	2.1546930	1.5300260
C	-2.8226900	-2.7833180	1.4331140
C	0.7393550	2.9127610	1.7679580
C	-3.1495250	-1.6436460	0.6296090
C	-0.3532690	2.8334840	0.8567010
C	-4.4853240	-1.1914420	0.5385040
C	-1.3351870	3.8528410	0.8073560
C	-4.8813040	-0.3436320	-0.4797200
C	-2.1814420	3.9694020	-0.2763200
C	-3.9509050	0.0194040	-1.4672410
C	-2.0261700	3.0904350	-1.3652760
C	4.3364850	1.0102250	0.3521330
C	4.1294320	-1.3938900	-1.0440100

C	5.3667380	-0.7885660	-0.8894520
C	5.4718830	0.4139650	-0.1728730
H	-1.9365450	-0.1568650	-2.1493570
H	-0.9613190	1.4248490	-2.1616390
H	2.6287600	-3.4191500	-0.4152680
H	0.6807670	-4.5562770	0.5665670
H	-1.4242890	-4.3702550	1.6987110
H	2.7461250	2.3050810	2.1221700
H	-3.5767260	-3.1994570	2.0961870
H	0.7277540	3.6614480	2.5557090
H	-5.2151270	-1.5833310	1.2431020
H	-1.3554650	4.5910010	1.6057310
H	-5.9165680	-0.0225780	-0.5557440
H	-2.9090810	4.7750150	-0.3218030
H	-4.2705210	0.5837750	-2.3387450
H	-2.5948560	3.2482940	-2.2775210
H	4.4236120	1.9697510	0.8514260
H	4.0513040	-2.3185170	-1.6083530
H	6.2512790	-1.2413870	-1.3288020
H	6.4394980	0.8931510	-0.0516510

50
ab-fused [6]helicene_-1230.375634 Hartree

C	-0.1776530	-3.5019080	1.8853250
C	0.8562990	-2.5865450	2.1289900
C	0.7624890	-1.2981340	1.6371590
C	-0.3478490	-0.8725430	0.8736890
C	-1.4331030	-1.7752690	0.7011470
C	-1.3073020	-3.0902990	1.2010200
C	-0.4364760	0.4937470	0.3467660
C	-1.7324230	0.9955200	0.0664940
C	-2.8451450	0.0974070	-0.0942430
C	-2.6808180	-1.3024600	0.1019970
C	0.6906410	1.3722200	0.1970560
C	0.4329530	2.7834420	0.2677450
C	-0.8756230	3.2678860	0.1160340
C	-1.9444260	2.4062160	-0.0962820
C	2.0725420	0.9596180	-0.0122930
C	3.1010870	1.8622520	0.3578680
C	2.7862710	3.2260180	0.6593830
C	1.5146740	3.6881920	0.5183480
C	2.4687640	-0.2769370	-0.6667660
C	3.8393060	-0.6855890	-0.6189170
C	4.8143870	0.1803040	-0.0368730
C	4.4635710	1.4311040	0.3768920
C	4.2273360	-1.9149030	-1.2071960
C	3.3175990	-2.6980130	-1.8847300
C	1.9865780	-2.2529130	-2.0200030
C	1.5775820	-1.0755850	-1.4281630
C	-4.1123940	0.6112220	-0.5085230
C	-5.1627390	-0.2782070	-0.7804690
C	-4.9742230	-1.6493480	-0.6515750
C	-3.7519270	-2.1539620	-0.2102170
C	-3.2562510	2.8909800	-0.4289380
C	-4.2854740	2.0334290	-0.6461720
H	-0.1124680	-4.5185560	2.2632470
H	1.7249980	-2.8802090	2.7114150
H	1.5560480	-0.5937790	1.8514910
H	-2.1283670	-3.7891300	1.0860360

H	-1.0446040	4.3424320	0.1284740
H	3.5983600	3.9006980	0.9186210
H	1.2875820	4.7451270	0.6325220
H	5.8494130	-0.1497770	0.0063680
H	5.2182210	2.1275490	0.7342990
H	5.2699700	-2.2164590	-1.1370640
H	3.6290280	-3.6349210	-2.3384130
H	1.2760270	-2.8354650	-2.5997800
H	0.5561750	-0.7457300	-1.5658510
H	-6.1216900	0.1176170	-1.1056550
H	-5.7848930	-2.3342520	-0.8846690
H	-3.6397050	-3.2278460	-0.1104360
H	-3.3970680	3.9644690	-0.5282180
H	-5.2665490	2.4065920	-0.9296820

50

TS_ab-fused [6]helicene_-1230.314986 Hartree_Imaginary frequency: -35.53 cm-1

C	-0.5371590	1.3016850	-0.6438500
C	0.5000500	0.2822980	-0.6256970
C	-1.8941180	1.1881240	-0.0622540
C	0.3500400	-1.1818850	-0.6633690
C	-2.7916610	0.0384350	-0.0048990
C	-0.4583430	-1.8138730	-1.6219890
C	-3.0380540	-0.8054840	-1.1097130
C	-0.0778370	2.6515360	-0.8915010
C	1.2683550	2.9352340	-1.1745030
C	-0.8990900	3.7890460	-0.5957380
C	2.2562900	2.0180410	-0.8545890
C	-1.9473360	3.6408760	0.2518030
C	1.8531180	0.6992830	-0.4776880
C	-2.3947300	2.3335720	0.6146740
C	2.8235480	-0.1589390	0.1584840
C	-3.3472000	2.1939640	1.6749090
C	2.4434330	-1.4375210	0.6514940
C	-3.8585070	0.9776140	2.0134530
C	1.2170680	-2.0128090	0.1030330
C	-3.6717410	-0.1180220	1.1166920
C	1.0027390	-3.4060520	0.0982590
C	-4.4908130	-1.2689450	1.2104260
C	0.0689190	-3.9906140	-0.7439420
C	-4.5904120	-2.1575520	0.1585350
C	-0.6145890	-3.1904690	-1.6655660
C	-3.9100020	-1.8758350	-1.0403510
C	3.3318790	-2.1393050	1.4764260
C	4.1465290	0.3203830	0.3956330
C	4.6082410	-1.6428910	1.7442520
C	5.0257820	-0.4425250	1.1813620
C	4.5434300	1.5921580	-0.1505320
C	3.6410590	2.3990310	-0.7629790
H	-0.9083840	-1.2008700	-2.3876700
H	-2.5965600	-0.5422180	-2.0590190
H	1.5482380	3.9560070	-1.4248580
H	-0.5511640	4.7744630	-0.8934840
H	-2.4672890	4.5022110	0.6635860
H	-3.6096470	3.0853960	2.2394110
H	-4.5169740	0.8593830	2.8700330
H	1.6387580	-4.0468670	0.6995920
H	-5.1038000	-1.3967240	2.0996330
H	-0.0634420	-5.0690570	-0.7439680

H	-5.2459380	-3.0212600	0.2281200
H	-1.2536580	-3.6394370	-2.4205100
H	-4.0802580	-2.4888070	-1.9211440
H	3.0371200	-3.0919740	1.9040940
H	5.2829090	-2.2090690	2.3805270
H	6.0300560	-0.0688370	1.3654660
H	5.5767530	1.9070410	-0.0274230
H	3.9299470	3.3792750	-1.1340380

50

cd-fused [6]helicene_-1230.37471 Hartree

C	4.4929040	-0.6922170	1.8567370
C	3.4172250	0.1805240	2.1014560
C	2.1851300	-0.0444680	1.5157990
C	1.9641820	-1.1436070	0.6525270
C	3.0333560	-2.0746790	0.4856690
C	4.2922030	-1.8108570	1.0714840
C	0.6811650	-1.4056520	0.0039510
C	0.4482830	-2.7328140	-0.4794730
C	1.5517580	-3.6443470	-0.6218510
C	2.8016190	-3.3070990	-0.2138360
C	-0.3845410	-0.4521400	-0.1172710
C	-1.7120220	-0.9481030	-0.2317390
C	-1.9454990	-2.3184770	-0.5848110
C	-0.8519830	-3.1483650	-0.8038900
C	-0.2161980	1.0025880	-0.1371880
C	-1.2788720	1.8169220	0.3075230
C	-2.6153440	1.2501260	0.4660730
C	-2.8290880	-0.0982050	0.0584980
C	0.9651100	1.6474220	-0.6851020
C	1.1701480	3.0426660	-0.4549290
C	0.1549460	3.7897480	0.2073610
C	-1.0422780	3.2115420	0.5152560
C	2.3448460	3.6740730	-0.9348960
C	3.2714370	2.9757280	-1.6774320
C	3.0309580	1.6206230	-1.9878170
C	1.9103570	0.9775330	-1.5053370
C	-4.1540280	-0.6313840	0.0188190
C	-4.3512150	-1.9901430	-0.4114350
C	-3.2970470	-2.7925950	-0.7081780
C	-3.7238880	2.0013760	0.8913130
C	-5.0113500	1.4681210	0.8720290
C	-5.2311210	0.1702900	0.4237910
H	5.4640560	-0.5038050	2.3061450
H	3.5492240	1.0355860	2.7585710
H	1.3683570	0.6315060	1.7345600
H	5.0984890	-2.5234650	0.9132170
H	1.3564550	-4.6151060	-1.0708380
H	3.6336050	-3.9947250	-0.3448240
H	-1.0098320	-4.1636340	-1.1610630
H	0.3211440	4.8467200	0.4008350
H	-1.8292010	3.8310660	0.9288600
H	2.4874120	4.7318520	-0.7258860
H	4.1657330	3.4699200	-2.0470320
H	3.7300080	1.0770370	-2.6172910
H	1.7416430	-0.0572040	-1.7738940
H	-5.3690630	-2.3669830	-0.4752700
H	-3.4534870	-3.8235520	-1.0159700
H	-3.5959600	3.0227430	1.2303930

H -5.8481290 2.0778540 1.2015880
H -6.2373680 -0.2406050 0.3992910
50
TS_cd-fused [6]helicene_-1230.31111 Hartree_Imaginary frequency: -36.41 cm-1
C 0.3614830 -0.4571440 -0.2399860
C -0.6827540 -1.4428850 -0.0126540
C 0.2288320 1.0114870 -0.0313530
C -2.1371290 -1.3299670 -0.1779110
C -0.9529780 1.8616030 -0.1671930
C -2.7379330 -0.7864960 -1.3274610
C -1.7908180 1.8469850 -1.3026610
C 1.7086820 -0.9637790 -0.2851420
C 1.9876030 -2.3694520 -0.2391990
C 2.8505720 -0.0975910 -0.2630730
C 1.0040000 -3.2050470 0.2681440
C 2.6898150 1.2376200 0.1775690
C -0.2849700 -2.7201230 0.5070490
C 1.3504810 1.6928450 0.4941480
C -1.1934840 -3.4836670 1.3270670
C 1.1637720 2.8211020 1.3562210
C -2.4616090 -3.0615120 1.5564030
C -0.0746540 3.3386640 1.5949080
C -3.0059730 -2.0273460 0.7200170
C -1.1522330 2.9469730 0.7479110
C -4.4028780 -1.8622910 0.6116270
C -2.3299730 3.7280910 0.6675330
C -4.9572860 -1.1759560 -0.4561540
C -3.2075420 3.5808150 -0.3878210
C -4.1143290 -0.7006360 -1.4711330
C -2.8885030 2.6795440 -1.4198060
C 3.8131600 2.0778200 0.2528860
C 4.1628180 -0.5946100 -0.5362100
C 5.2540940 0.2851550 -0.4895860
C 5.0760690 1.6116320 -0.1065840
C 3.3040770 -2.8569920 -0.5555120
C 4.3388050 -2.0034160 -0.7605580
H -2.0990610 -0.5089730 -2.1521870
H -1.5014650 1.2319820 -2.1407930
H 1.2456270 -4.2331680 0.5288650
H -0.8023940 -4.3742160 1.8126460
H 2.0253990 3.2045210 1.8924800
H -3.1184020 -3.5879430 2.2439870
H -0.2242370 4.1302490 2.3244130
H -5.0428060 -2.3564450 1.3389420
H -2.4872280 4.5053840 1.4116850
H -6.0357140 -1.0781910 -0.5454550
H -4.0923450 4.2072850 -0.4598310
H -4.5349350 -0.2761510 -2.3783930
H -3.4951800 2.6447060 -2.3204180
H 3.6941340 3.1141250 0.5510810
H 6.2465660 -0.0898210 -0.7270100
H 5.9276310 2.2857250 -0.0729750
H 3.4482720 -3.9332120 -0.6103590
H 5.3308380 -2.3744080 -1.0055810
58
abcd-fused [6]helicene_-1460.264035 Hartree
C 2.7527730 2.9684540 2.2764030
C 1.3577450 2.8753610 2.3744160

C	0.6934750	1.8381710	1.7451830
C	1.3835790	0.8721380	0.9801300
C	2.8015560	0.9261000	0.9414990
C	3.4571500	1.9955620	1.5877700
C	0.6707710	-0.2235880	0.3140110
C	1.4093700	-1.4081990	0.0089070
C	2.8465530	-1.3520740	-0.0487780
C	3.5488350	-0.1635980	0.3083070
C	-0.7137290	-0.1962790	0.0088720
C	-1.4063180	-1.4474260	-0.0482930
C	-0.6871880	-2.6672030	-0.1849630
C	0.7345540	-2.6187510	-0.3156980
C	-1.4922990	1.0201920	-0.2338500
C	-2.8706060	1.0146740	0.0547940
C	-3.5580460	-0.2539910	0.2995160
C	-2.8314260	-1.4688100	0.1176690
C	-0.9111090	2.2065060	-0.8363130
C	-1.6537610	3.4276840	-0.8275760
C	-2.9880160	3.4205040	-0.3334840
C	-3.5881690	2.2492910	0.0316510
C	-1.0783590	4.6100010	-1.3576750
C	0.1697040	4.5905310	-1.9394710
C	0.8707250	3.3692400	-2.0357050
C	0.3441330	2.2128580	-1.5003540
C	-3.5159970	-2.7196940	0.1785730
C	-2.7684310	-3.9249940	-0.0043550
C	-1.4180400	-3.8983550	-0.1838690
C	-4.9247050	-0.3343330	0.5982380
C	-5.5848260	-1.5638520	0.6759340
C	-4.8958390	-2.7453120	0.4532070
C	3.5764770	-2.4842450	-0.5220070
C	4.9722900	-2.3973470	-0.6824910
C	5.6372710	-1.2182680	-0.3883910
C	4.9329410	-0.1162610	0.1056370
C	1.5075980	-3.7483920	-0.7343820
C	2.8634320	-3.6802410	-0.8482990
H	3.2855840	3.7783260	2.7671780
H	0.7969830	3.6038490	2.9531500
H	-0.3816340	1.7597900	1.8488110
H	4.5403860	2.0451860	1.5816740
H	-3.5462200	4.3533780	-0.3136580
H	-4.6343390	2.2664450	0.3131490
H	-1.6556370	5.5308660	-1.3173230
H	0.6003680	5.5003150	-2.3486530
H	1.8314740	3.3348450	-2.5418420
H	0.8941800	1.2867920	-1.6065900
H	-3.2983000	-4.8737450	0.0295890
H	-0.8821410	-4.8359170	-0.2796470
H	-5.5024180	0.5674940	0.7637110
H	-6.6466570	-1.5865790	0.9052490
H	-5.4073400	-3.7031010	0.5067980
H	5.5163810	-3.2617810	-1.0548450
H	6.7117860	-1.1464670	-0.5328440
H	5.4835900	0.7901790	0.3308450
H	1.0019070	-4.6669260	-1.0103730
H	3.4276320	-4.5392810	-1.2029490

58
TS_abcd-fused [6]helicene_-1460.201349 Hartree_Imaginary frequency: -29.43 cm-1

C	0. 6521180	-0. 0847390	-0. 4566840
C	-0. 7770870	-0. 1535290	-0. 5251860
C	1. 4448330	1. 0635990	0. 0603500
C	-1. 7520310	0. 9376910	-0. 7037180
C	1. 1691190	2. 4918490	-0. 0336750
C	-1. 6371850	1. 8665650	-1. 7486400
C	0. 8129510	3. 1280370	-1. 2440150
C	1. 3562600	-1. 3476770	-0. 5536160
C	0. 6856300	-2. 5549210	-0. 8886320
C	2. 7503720	-1. 4517240	-0. 2351660
C	-0. 7092630	-2. 6316610	-0. 6116390
C	3. 3537180	-0. 4365790	0. 5494640
C	-1. 4017270	-1. 4310100	-0. 3216340
C	2. 5744000	0. 7539680	0. 8490020
C	-2. 7064340	-1. 5013760	0. 2877650
C	2. 9583410	1. 6300540	1. 9120900
C	-3. 3935250	-0. 3156170	0. 6768280
C	2. 3473770	2. 8366920	2. 0978710
C	-2. 9724310	0. 9276060	0. 0278200
C	1. 5229640	3. 3525820	1. 0566690
C	-3. 8567850	2. 0142210	-0. 1098270
C	1. 2066480	4. 7316130	0. 9929990
C	-3. 6288510	3. 0168640	-1. 0426860
C	0. 7269510	5. 2949540	-0. 1717930
C	-2. 5463780	2. 9010720	-1. 9187740
C	0. 6008070	4. 4911940	-1. 3211210
C	4. 6919740	-0. 5783860	0. 9384240
C	3. 4959320	-2. 6221790	-0. 5667740
C	4. 8511110	-2. 7064220	-0. 1977410
C	5. 4384060	-1. 6937830	0. 5473290
C	1. 4553730	-3. 6909530	-1. 3049210
C	2. 8135620	-3. 7055230	-1. 2044990
C	-1. 3835230	-3. 8857560	-0. 4456090
C	-3. 2742480	-2. 7709890	0. 5991810
C	-4. 5280410	-0. 4234000	1. 4840350
C	-5. 0489880	-1. 6745420	1. 8357000
C	-4. 4536920	-2. 8347900	1. 3677170
C	-2. 6072510	-3. 9540730	0. 1487550
H	-0. 8510400	1. 7217840	-2. 4737980
H	0. 8009580	2. 5357100	-2. 1476270
H	3. 7026760	1. 2840460	2. 6217760
H	2. 5816660	3. 4597610	2. 9569620
H	-4. 7804020	2. 0285040	0. 4591790
H	1. 4272750	5. 3538840	1. 8571430
H	-4. 3355050	3. 8360460	-1. 1433810
H	0. 5205290	6. 3604700	-0. 2229190
H	-2. 4176830	3. 6058380	-2. 7351750
H	0. 3509400	4. 9435720	-2. 2768720
H	5. 1751870	0. 2055060	1. 5118640
H	5. 4215790	-3. 5885360	-0. 4775770
H	6. 4830980	-1. 7673500	0. 8369580
H	0. 9386530	-4. 5550130	-1. 7095450
H	3. 3852930	-4. 5706270	-1. 5307390
H	-0. 8725800	-4. 8039150	-0. 7128840
H	-5. 0324980	0. 4724580	1. 8312620
H	-5. 9375090	-1. 7299970	2. 4585740
H	-4. 8735930	-3. 8079120	1. 6099550
H	-3. 0754970	-4. 9178270	0. 3329470

64
abcde-fused [6]helicene_-1613.907741 Hartree

C	1.5990720	-3.9056550	-1.9524600
C	0.3830840	-3.2154440	-2.0463750
C	0.2758140	-1.9400490	-1.5219950
C	1.3600120	-1.3118370	-0.8684160
C	2.6125880	-1.9805400	-0.8462270
C	2.6959140	-3.2839530	-1.3804460
C	1.2397900	0.0405000	-0.3125350
C	2.4361370	0.8029830	-0.1526240
C	3.7083700	0.1287230	-0.1244870
C	3.7989730	-1.2750710	-0.3562980
C	0.0011410	0.6508390	0.0174020
C	-0.0769170	2.0775940	-0.0003270
C	1.1089150	2.8659930	-0.0041730
C	2.3757550	2.2106680	0.0586920
C	-1.2145880	-0.0959770	0.3239050
C	-2.4543280	0.4694850	0.0161710
C	-2.5598890	1.9244070	-0.0443390
C	-1.3636320	2.7088020	-0.0086080
C	-1.1942970	-1.3694180	1.0436590
C	-2.3636970	-2.1749510	1.0853750
C	-3.5180180	-1.7718470	0.2925390
C	-3.5752560	-0.4412240	-0.2128390
C	-2.3638040	-3.3410410	1.8801510
C	-1.2511610	-3.7138370	2.6132370
C	-0.1021660	-2.9085680	2.5898810
C	-0.0805760	-1.7576200	1.8244670
C	-1.4490830	4.1337210	0.0009850
C	-0.2400440	4.8971780	-0.0228120
C	0.9800620	4.2918260	-0.0300080
C	-3.7899360	2.5991800	0.0522160
C	-3.8622010	3.9935470	0.0832060
C	-2.7084750	4.7590940	0.0281680
C	4.8887930	0.8640240	0.1981430
C	6.1164510	0.1899730	0.3350890
C	6.1818790	-1.1836860	0.1661260
C	5.0363840	-1.9058850	-0.1792870
C	3.5920420	2.9192900	0.3238100
C	4.7896400	2.2760750	0.4029100
C	-4.5647560	-2.6660080	-0.0148690
C	-5.6310450	-2.2882440	-0.8129200
C	-5.6607770	-0.9987500	-1.3624580
C	-4.6486020	-0.1000050	-1.0716520
H	1.6952910	-4.9084400	-2.3596280
H	-0.4712130	-3.6688020	-2.5410200
H	-0.6592740	-1.4048360	-1.6310910
H	3.6471150	-3.8044720	-1.3826990
H	-3.2625580	-3.9452830	1.9450510
H	-1.2782880	-4.6135340	3.2218590
H	0.7661370	-3.1750660	3.1859330
H	0.7990050	-1.1263670	1.8442700
H	-0.3136280	5.9817010	-0.0431280
H	1.8709960	4.9077350	-0.0732850
H	-4.7062660	2.0332450	0.1563840
H	-4.8337320	4.4742490	0.1587590
H	-2.7615910	5.8449110	0.0290880
H	7.0064220	0.7593870	0.5912510

H	7.1262780	-1.7055750	0.2939880
H	5.1217570	-2.9790100	-0.3087520
H	3.5575670	3.9866510	0.5094050
H	5.6947820	2.8301150	0.6390990
H	-4.5168860	-3.6873310	0.3473020
H	-6.4183380	-3.0012740	-1.0414510
H	-6.4601910	-0.7092720	-2.0389940
H	-4.6487910	0.8655030	-1.5622020

64

TS_abcde-fused [6]helicene_-1613.852449 Hartree_Imaginary frequency: -24.29 cm-1

C	-0.0824950	0.4867980	-0.7723080
C	1.2888760	0.0917350	-0.6426850
C	-1.2581040	-0.3807650	-0.5316120
C	1.9002390	-1.2154550	-0.9254040
C	-1.4164970	-1.8251290	-0.7591190
C	1.6533440	-1.8927130	-2.1301570
C	-1.1232330	-2.4007620	-2.0100140
C	-0.3369410	1.9117740	-0.7740050
C	0.7142220	2.8659770	-0.7567670
C	-1.6764450	2.4124340	-0.7558050
C	1.9753100	2.4441530	-0.2461940
C	-2.7221070	1.5688870	-0.2966640
C	2.2160760	1.0530740	-0.1172430
C	-2.3781310	0.2070590	0.0734990
C	3.3600120	0.6022530	0.6353430
C	-3.0959220	-0.5475090	1.0997580
C	3.6021850	-0.7878940	0.8326280
C	-2.8529140	-1.9421130	1.2509220
C	2.9455140	-1.7090830	-0.0968880
C	-2.1558710	-2.6154710	0.1646670
C	3.4773140	-2.9854530	-0.3627410
C	-2.3310870	-3.9903020	-0.0925990
C	3.0951740	-3.7075370	-1.4849270
C	-1.9023150	-4.5613320	-1.2800760
C	2.2255130	-3.1244000	-2.4117510
C	-1.3479710	-3.7424320	-2.2717860
C	-4.0385350	2.0545400	-0.3064870
C	-1.9548760	3.7740410	-1.0766070
C	-3.2895380	4.2196290	-1.0860570
C	-4.3175290	3.3588140	-0.7255940
C	0.4257760	4.2263380	-1.1042270
C	-0.8513610	4.6510370	-1.3213870
C	2.9379480	3.3716860	0.2682670
C	4.2086010	1.5560510	1.2698390
C	4.5719300	-1.1822810	1.7575330
C	5.3633210	-0.2384310	2.4225040
C	5.2104620	1.1126890	2.1560670
C	3.9985730	2.9476870	1.0110730
C	-3.8578390	0.1081300	2.0961230
C	-3.3661390	-2.6173140	2.3731270
C	-4.1218070	-1.9541180	3.3275870
C	-4.3682140	-0.5815940	3.1845430
H	1.0525080	-1.3964030	-2.8779310
H	-0.7905650	-1.7535690	-2.8080100
H	4.2625240	-3.3813260	0.2726660
H	-2.8832200	-4.5977350	0.6171810
H	3.5338300	-4.6825880	-1.6782860
H	-2.0671300	-5.6188790	-1.4669180

H	2.0072290	-3.6210980	-3.3527870
H	-1.1149190	-4.1472040	-3.2527560
H	-4.8530750	1.3982080	-0.0208060
H	-3.5021790	5.2493200	-1.3624120
H	-5.3480800	3.7023440	-0.7503390
H	1.2486110	4.9245370	-1.2179960
H	-1.0512540	5.6788510	-1.6136980
H	2.7708470	4.4355070	0.1403460
H	4.7333350	-2.2368710	1.9560110
H	6.1145990	-0.5728820	3.1326040
H	5.8449940	1.8475260	2.6453010
H	4.6817610	3.6679380	1.4542890
H	-3.9971670	1.1810020	2.0417100
H	-3.1506170	-3.6731310	2.5042990
H	-4.5040900	-2.4934200	4.1898540
H	-4.9350500	-0.0463220	3.9414210
